# An In Vitro Evaluation and Network Pharmacology Analysis of Prospective Anti-Prostate Cancer Activity from *Perilla frutescens*

**DOI:** 10.3390/plants12163006

**Published:** 2023-08-21

**Authors:** Patrick Jay B. Garcia, Steven Kuan-Hua Huang, Kathlia A. De Castro-Cruz, Rhoda B. Leron, Po-Wei Tsai

**Affiliations:** 1 School of Chemical, Biological, and Materials Engineering and Sciences, Mapúa University, Intramuros, Manila 1002, Philippines; pjbgarcia@mymail.mapua.edu.ph (P.J.B.G.); kadecastro@mapua.edu.ph (K.A.D.C.-C.); rbleron@mapua.edu.ph (R.B.L.); 2 School of Graduate Studies, Mapúa University, Intramuros, Manila 1002, Philippines; 3 Department of Medical Science Industries, College of Health Sciences, Chang Jung Christian University, Tainan 711, Taiwan; 7224837@mail.cjcu.edu.tw; 4 Division of Urology, Department of Surgery, Chi Mei Medical Center, Tainan 711, Taiwan; 5 School of Medicine, College of Medicine, Kaohsiung Medical University, Kaohsiung 807, Taiwan

**Keywords:** *Perilla frutescens*, green perilla, phytochemical content, in vitro, prostate cancer, in silico, network pharmacology

## Abstract

*Perilla frutescens* (L.) Britt. is extensively cultivated in East Asia as a dietary vegetable, and nutraceuticals are reportedly rich in bioactive compounds, especially with anticancer activities. This study explored the in vitro cytotoxic effects of *P. frutescens* parts’ (stems, leaves, and seeds) extracts on prostate cancer cells (DU-145) and possible interactions of putative metabolites to related prostate cancer targets in silico. The ethanol extract of *P. frutescens* leaves was the most cytotoxic for the prostate cancer cells. From high-performance liquid chromatography analysis, rosmarinic acid was identified as the major metabolite in the leaf extracts. Network analysis revealed interactions from multiple affected targets and pathways of the metabolites. From gene ontology enrichment analysis, *P. frutescens* leaf metabolites could significantly affect 14 molecular functions and 12 biological processes in five cellular components. Four (4) KEGG pathways, including for prostate cancer, and six (6) Reactome pathways were shown to be significantly affected. The molecular simulation confirmed the interactions of relevant protein targets with key metabolites, including rosmarinic acid. This study could potentially lead to further exploration of *P. frutescens* leaves or their metabolites for prostate cancer treatment and prevention.

## 1. Introduction

Prostate cancer (PCa) affected 14.1% of men worldwide, particularly old-aged men, in 2020 [1]. In this report, it is the fifth most death-causing cancer disease. Most PCa cells are potentially metastatic and are sometimes detectable from the emergence of urethral blockage or hematuria [2]. These are usually late-stage manifestations that allude to appropriate monitoring, especially for high-risk individuals. The early detection of PCa is currently tested with prostate-specific antigen (PSA) levels in blood serum [3]. However, it is reported to have broad-ranged screening specificity and sensitivity, and PSA levels are increased for patients with benign prostate hyperplasia (BPH), which is usually a noncancerous prostate gland enlargement [4]. For this reason, additional tests such as biopsy or the detection of other biomarkers are required. Current non-metastatic hormone-sensitive PCa (nmHSPC) treatments are hormonal therapy, radiotherapy, immunotherapy, or castration/orchiectomy [3,5]. However, metastatic HSPC (mHSPC) and castrate-resistant PCa (mCRPC), which are advanced PCa, are unresponsive to such hormonal therapy and surgical procedures, which requires life-maintaining treatment and palliative care since they are generally incurable [5,6]. It is a wide-ranging disease that follows particular cell growth and progression mechanisms with complex molecular pathogenesis and etiology, which require multi-targeted treatments [5].

An increasing number of investigations are being conducted of PCa for traditional and complementary medicines, solely or in decoctions, as supplemental to such treatments or as a preventative measure [7,8,9]. These medicinal systems are commonly validated with non-physical mechanisms and alternative anatomical interpretations, which are viewed in obscurity and discord with biological mechanisms to some extent. Current efforts in traditional and complementary medicine research to uncover molecular mechanisms and therapeutic effects have increased the confidence of health consumers in this medicinal system [7,10,11,12]. Still, natural products have been a great source of biologically active molecules in conventional medicine, such as the chemotherapeutic drug Paclitaxel and Taxifolin from *Taxus chinensis* (Chinese yew) [8,11,13]. While plant materials may be low in concentration of these bioactive molecules compared to current medications, potent plant materials and extracts have been discovered to be indicative of excellent hit compounds [14,15]. Previously, it was reported that conjunctive use of traditional Chinese medicine (TCM), especially the Chai-Hu-Jia-Long-Gu-Mu-Li-Tang decoction, increased the survivability of metastatic PCa patients [16].

One of these traditional medicines is the *Perilla frutescens* (L.) Britt., which is an annual plant native and widely cultivated in eastern, southeastern, and southern Asia, mainly in subtropical climates, whereas it was introduced in northeastern America and southern Europe [17]. This plant has excellent nutraceutical and pharmaceutical value in TCM, and its leaves are a staple food in East Asian cuisine [18]. This plant has been traditionally used for but is not limited to athlete’s foot, generalized edema, cough, antibiotic, antidepressants, anti-lung, and colon cancer [19]. In fact, Jeong et al. [20] confirmed that ethanol extracts of this plant inhibited the metastatic ability of cancer cells, specifically breast and liver cancer cells, through Src kinase deactivation, thereby blocking the epithelial–mesenchymal transition (EMT) process. Also, a study by Lee et al. [21] uncovered the activity of isoegomaketone, an isolate in *P. frutescens* oils, as an inducer of apoptotic mechanisms on PCa cells from the activation of death receptors 4 and 5. Additionally, numerous secondary metabolites from plants’ adaptive mechanisms in different conditions could be a good source of compounds for multiple targets.

Network pharmacology is the current paradigm of molecular investigations of herb and herbal formulae in traditional medicines [22,23,24]. This computational approach includes the chemical diversity of TCMs into multiple targets through experimental data and predictive models aimed at advances in polypharmacology and multi-targeted drug discovery approach [23,24]. This network-driven approach overcomes the barriers of the conventional one-drug-one-target scheme and, therefore, is appropriate for complex diseases, mainly due to the diversity of cancer mechanisms. Based on the common targets between the disease and active compounds from the TCM herbals, this network-based method can characterize potential key molecular targets and mechanisms of action.

The abundance of compounds with anticancer activities in various parts of *P. frutescens* var. *frutescens* (PF), the green variety, has potential applications in PCa treatment or prevention [25]. Furthermore, this study focused on polar compounds, including phenolic and flavonoid compounds, which are abundant in water and ethanol extracts of *P. frutescens* and commonly have anticancer properties [26,27,28,29]. The cytotoxic effects of these extracts were explored with prostate adenocarcinoma (PRAD) DU-145 cell lines. The metabolites’ action was briefly studied with in silico network pharmacology. There is limited information about their potential anti-PCa activity; hence, this study addressed initial interests in the bioactivity of the plant’s extracts as supplemental to PCa treatment or its prevention. Also, understanding the molecular mechanism of the plant could potentially decipher drug-to-herb interaction as a necessary criterion for their adjuvant or concomitant use with conventional medicines.

## 2. Results

### 2.1. Total Phytochemical Content

The apparent phytochemical contents of the PF parts relevant to anticancer activity were assessed from total phenolics and flavonoid contents as shown in Table 1. The leaf extracts revealed superior amounts of phytochemicals compared to the other studied parts. For the total phenolics content with a Folin–Ciocâlteu assay, PF-L-W > PF-L-E > PF-SD-W > PF-S-E > PF-S-W > PF-SD-E, whereas for total flavonoid content with an AlCl_3_ assay, PF-L-W > PF-L-E > PF-SD-E > PF-S-E > PF-SD-W. PF leaves (PF-L) and water extracts were observed to obtain the highest amount of such phytochemicals.

### 2.2. Anti-Prostate Cancer Activity

**Figure 1:**
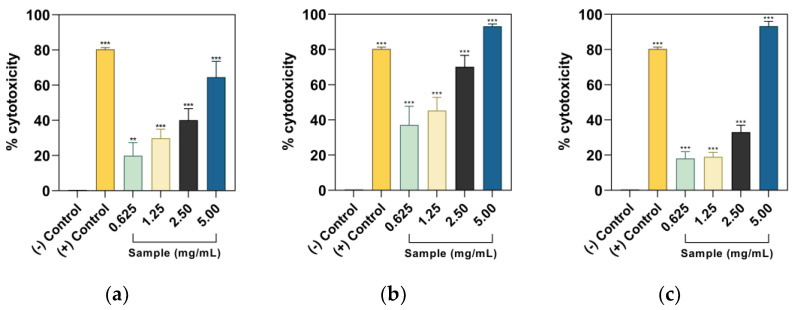

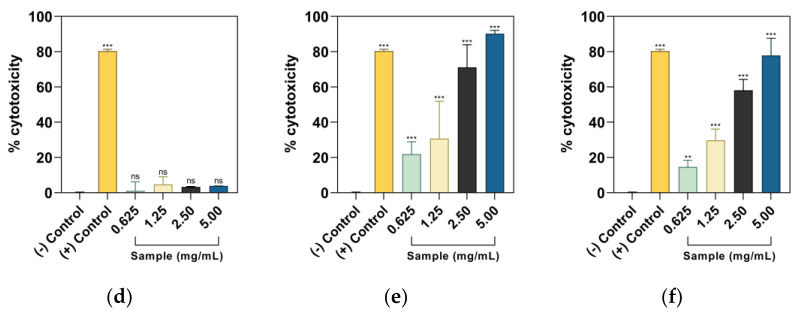
Concentration–cytotoxicity curves of PF parts crude extracts, i.e., ethanol extracts of (**a**) seeds, (**b**) leaves, (**c**) seeds, and water extracts of (**d**) stems, (**e**) leaves, and (**f**) seeds. The negative (−) control for ethanol extract contains the media and added DMSO, whereas water extract only includes the media. Comparisons with the negative control ** *p* < 0.01, *** *p* < 0.001, and *ns* are not significant according to Dunnett’s pairwise comparison test.

Dose–response behavior was observed for all samples; however, the water extract of PF seeds was not significantly different from the negative control and had no dose–response behavior. In Figure 1, the 5.00 mg/mL dose of ethanolic PF leaf and seeds and aqueous leaf extracts surpassed the cytotoxicity of 5-fluorouracil (5FU). The highest half-maximal cytotoxic concentrations were observed from PF-L-E (1.3982 ± 0.3453 mg/mL) > PF-L-W (1.6224 ± 0.2926 mg/mL) > PF-SD-W (2.0612 ± 0.0327 mg/mL) > PF-SD-E (2.1631 ± 0.7595 mg/mL) > PF-S-E (3.5275 ± 0.4574 mg/mL). The PF part extract with the most cytotoxic potential of DU-145 cells is found in their leaves, specifically for the ethanol extract.

### 2.3. Detection of the Major Compound

**Figure 2:**
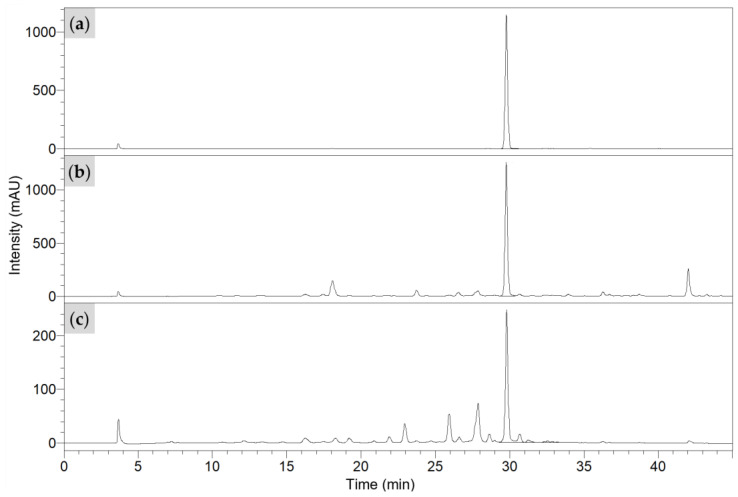
HPLC chromatogram of (**a**) 500 µg/Ml rosmarinic acid standard, (**b**) ethanol extract, and (**c**) water extract of PF-L.

Metabolomic analyses of PF-L show that its major metabolite is rosmarinic acid (RA), a phenolic acid [30,31]. For verification, the presence of rosmarinic acid in the crude PF-L extracts was evaluated with HPLC analysis. Peak identification revealed its retention time at *t_R_* = 29.82 min in Figure 2.

### 2.4. Network Pharmacology

**Figure 3:**
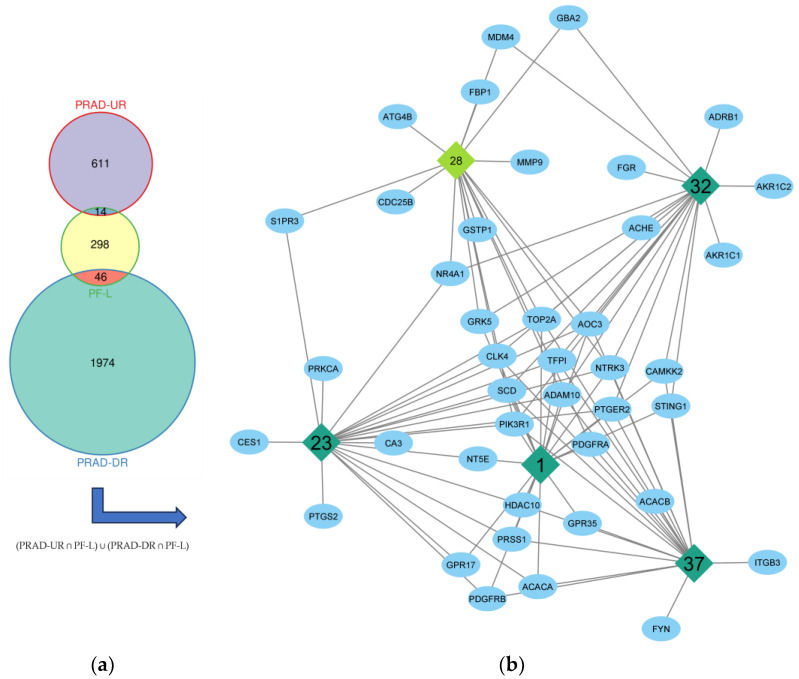
(**a**) Intersected genes set from predicted targets of PF-L metabolites and expressed/overexpressed genes of PRAD compared to normal prostate cells where DR are downregulated genes and UR are upregulated genes; (**b**) key metabolites (including RA) in the MTN of PF-L against PRAD.

Putative metabolites of PF-L were collected from the literature as summarized in Table S1, excluding the nonpolar compounds [30,31,32,33]. There were 358 predicted targets of these metabolites after duplicate removal. From differentially expressed genes of PRAD versus normal prostate cells, 14 upregulated and 46 downregulated genes are associated with cytotoxicity of PF-L metabolites to DU-145 cells, shown in Figure 3a. The higher degree of node connections’ strength and amount in these network analyses indicates its importance. These nodes with higher connection degrees could potentially affect multiple targets simultaneously. In the metabolite-target network (MTN) for these common targets in Figure S1, simplified in Figure 3b, with at least 20 affected targets were tuberonic acid (**37**, 20 targets), scutellarein-7-*O*-glucuronide (**32**, 20 targets), 5’-gluco-pyranosyoxyjasmanic acid or tuberonic acid glucoside (**1**, 20 targets), and *n*-octanoylsucrose (**23**, 21 targets). The major compound, RA (28), in this MTN analysis had 17 common targets.

**Figure 4:**
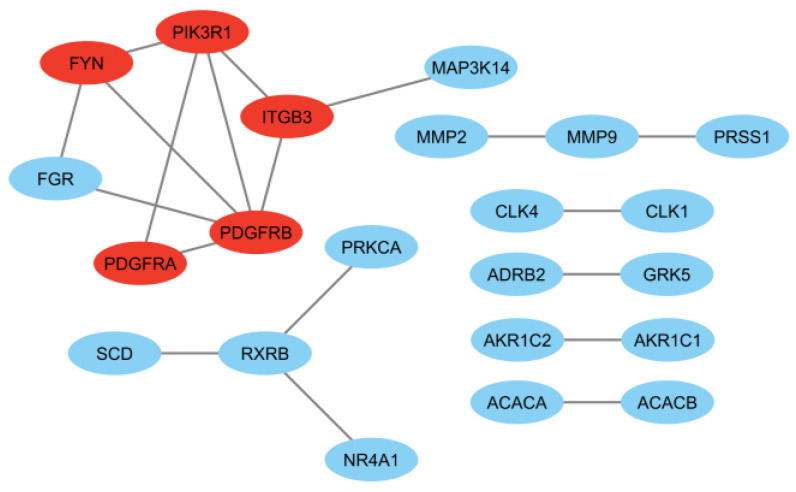
PPI network of intersected genes set with interaction score of at least 0.90 where red-labeled proteins are top-ranking in interactivity through MCC and DMNC analysis.

Interaction of the proteins from the protein-coding genes seen in Figure S2 revealed a highly significant network (*p* = 9.04 × 10^−12^) with 76 connections, an average node degree of 2.53, an average local clustering coefficient of 0.382, and a maximum interaction score of 0.50. An increased required interaction score of 0.90 revealed a significant network (*p* = 0.00033) with 19 significant interactions, an average node degree of 0.633, and an average local clustering coefficient of 0.298 (Figure 4). Henceforth, the top five (5) targets from topological analysis in Cytoscape with maximal clique centrality (MCC) and density of maximum neighborhood component (DMNC) algorithms were considered key targets for PCa cytotoxicity of PF-L metabolites. The MCC and DMNC algorithms were previously observed to best identify essential targets in a complex interactome [34]. In this analysis, the p59-Fyn proto-oncogene (FYN), platelet-derived growth factor receptors α and β (PDGFRA and PDGFRB), phosphoinositide-3-kinase regulatory subunit 1 (PIK3R1), and integrin α_V_β_3_ (ITGB3) were top-ranking. Based on the network in Figure S3, metabolites with at least three (3) affected PPI network-relevant targets were 5′-gluco-pyranosyoxyjasmanic acid (**1**, 3 targets), apigenin-7-*O*-diglucuronide (**5**, 3 targets), coumaric acid-4-*O*-glucoside (**15**, 3 targets), perillaldehyde (**24**, 3 targets), trans-*p*-menth-8-en-yl caffeate (**36**, 4 targets), and tuberonic acid (**37**, 5 targets). From these analyses, compounds **1** and **37** were defined for both MTN.

The gene ontology (GO) terms classified expressed properties from genes and their products according to their molecular functions (MF), involvement in biological processes (BP), and presence in cellular components (CC). In Figure S4, GO terms and pathways were ordered according to the adjusted *p*-value from the g:SCS algorithm. A higher *p*-value indicates that it has a significant PCa cytotoxicity effect from the metabolite action on protein-coding gene targets. According to the enriched GO terms, 14 MFs, 12 BPs, and 5 CCs were considered significant from 60 intersecting gene targets. Biological processes relevant to the PCa cytotoxic effect of PF-L metabolites are the responses to oxygen-containing compounds, protein phosphorylation, and cellular component disassembly, mainly distributed in the cytoplasm, cell surface, and periphery. These results reveal that the metabolites may influence the activity of ions, protein kinase, and identical protein binding.

The apparent mechanism of action of PF-L activity on PCa may be deciphered from significant pathways from KEGG and Reactome. Furthermore, pathway enrichment analysis showed four (4) KEGG pathways and six (6) Reactome pathways, which involved 28 targets, which are acetyl-CoA carboxylase 1 (ACACA), acetyl-CoA carboxylase 2 (ACACB), acetylcholinesterase (ACHE), disintegrin and metalloproteinase domain-containing protein 10 (ADAM10), aldose reductase (AKR1B1), 20α/3α-hydroxysteroid/dihydrodiol dehydrogenase (AKR1C1), 3α-hydroxysteroid 3-dehydrogenase (AKR1C2), tyrosine-protein kinase receptor UFO (AXL), calcium/calmodulin-dependent protein kinase kinase 2 (CAMKK2), M-phase inducer phosphatase 2 (CDC25B), fructose-1,6-bisphosphatase 1 (FBP1), tyrosine-protein kinase Fyn (FYN), non-lysosomal glucosylceramidase (GBA2), glutathione S-transferase P (GSTP1), cortisone reductase (HSD11B1), integrin α_V_β_3_ (ITGB3), protein Mdm4 (MDM4), gelatinase A (MMP2), gelatinase B (MMP9), nuclear receptor 4A1 (NR4A1), platelet-derived growth factor receptor α (PDGFRA), platelet-derived growth factor receptor β (PDGFRB), phosphoinositide-3-kinase regulatory subunit 1 (PIK3R1), membrane associated phospholipase A2 (PLA2G2A), protein kinase C-alpha (PRKCA), prostaglandin G/H synthase 2 (PTGS2), retinoic acid receptor RXR-β (RXRB), and stearoyl-CoA desaturase (SCD). The first two pathways were cell growth and proliferation-related pathways; there are three (3) cancer-related pathways, two (2) metabolism-related pathways, a cell segregation-related pathway, and an angiogenesis-related pathway. Based on the protein targets affected visualized in Figures S5–S14, PF-L metabolites have diverse targets where their activities are still in need of further validation and research.

### 2.5. Molecular Docking

**Figure 5:**
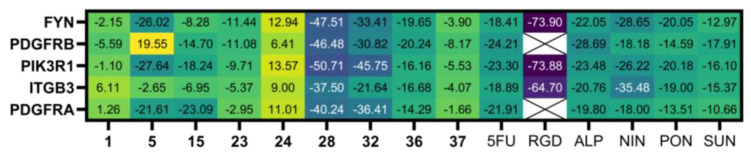
CDOCKER energy of protein–ligand interaction (in kcal/mol) where lower energy (darker color) indicates good binding affinity. Controls’ abbreviations: 5FU, 5-fluorouracil; RGD, [RGD-ChgE]-CONH_2_; ALP, alpelisib; NIN, nintedanib; PON, ponatinib; SUN, sunitinib.

**Figure 6:**
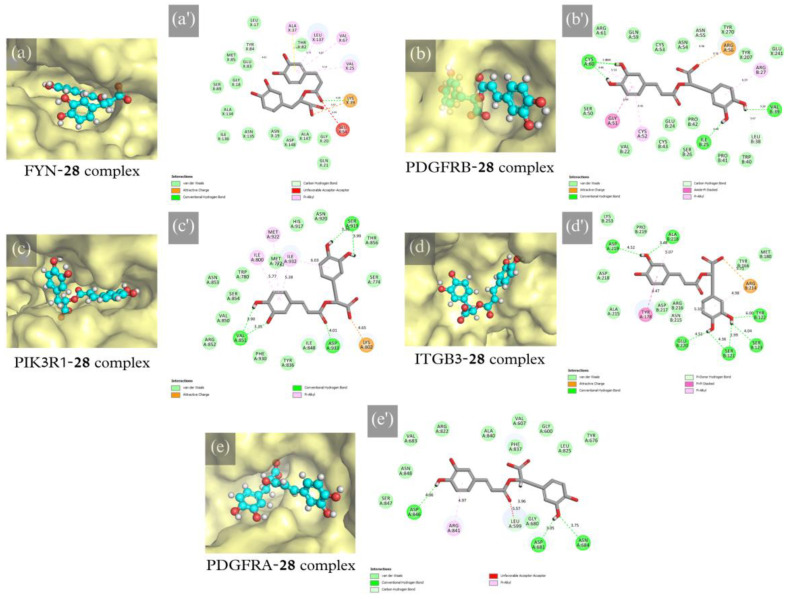
(**a**–**e**) Protein–ligand binding site and (**a′**–**e′**) intermolecular interactions of docked RA (**28**) with (**a**,**a′**) FYN, (**b**,**b′**) PDGFRB, (**c**,**c′**) PIK3R1, (**d**,**d′**) ITGB3, and (**e**,**e′**) PDGFRA.

For protein–ligand interaction validation, selected protein targets and metabolites from network pharmacology results were subjected to molecular docking. The binding site was selected with the volume of co-crystallized ligands for FYN (PDB code: 2DQ7), PIK3R1 (PDB code: 4JPS), ITGB3 (PDB code: 6MK0), and PDGFRA (PDB code: 6JOK). At the same time, the most significant volume from the grid-search and eraser algorithm was determined for PDGFRB (PDB code: 3MJG). Controls used were 5-fluorouracil (CID: 3385), [RGD-ChgE]-CONH_2_ [35], alpelisib (CID: 56649450), nintedanib (CID: 135423438), ponatinib (CID: 24826799), and sunitinib (CID: 5329102). The highest docking score from Table S5 for each ligand was selected, shown in Figure 5. RA (**28**) has the best docking score compared to 5FU. For metabolites in network pharmacology, all proteins were most stably docked with scutellarein-7-*O*-glucuronide (SG, **32**). These compounds were more stable and spontaneously binding than the positive controls except for [RGD-ChgE]-CONH_2_. Prevalent drug–receptor interactions, such as hydrophobic contact, hydrogen bonding, and π-stacked interactions [36], were observed in all protein-**28** complexes shown in Figure 6.

## 3. Discussion

PF has a wide array of pharmacologically active compounds historically used in TCM. Phytochemicals, such as polyphenols, are secondary metabolites of plants that are generally responsible for their defense mechanism against external stresses, such as infection, predators, environmental changes, and, to some extent, their growth [37]. Most polyphenols are anticancer agents targeting apoptotic and cell cycle pathways, along with cancer cell proliferation, tumorigenesis, angiogenesis, and metastatic abilities [26,27,28,29]. Plant extracts are an excellent source of these cancer-curative and preventative compounds due to their diverse targets and potentially synergistic action against cancer. Since PF-L had the most total phenolic and flavonoid content, its anti-PCa activity could be superior to other parts of PF.

Cancer is a multifactorial disease triggered through genetic alterations and instabilities, epigenetics, weakened immunosurveillance, infection, and so on [38]. Hallmarks of cancer pathogenesis revolve around the heightened activity of oncogenes (like growth-related genes responsible for tumor proliferation) and contrarywise for tumor suppressor genes [39]. A multi-targeted approach is appropriate since this disease takes advantage of each cell type’s specific cellular and metabolic machinery. The development of PCa at every stage has apparent changes in cellular mechanisms [40]. Henceforth, numerous in vitro models for PCa, such as the DU-145, PC-3, and LNCaP cell lines, are utilized for anti-prostate cancer activity research [41,42]. The prostate adenocarcinoma DU-145 cells, which are castration-resistant prostate adenocarcinoma from central nervous system (CNS) metastasis, were utilized for screening in this study [43]. While it is a negative-androgen receptor-expressing cell line, current reports reveal that DU-145 has positive androgen receptor expression in mediocre levels compared to androgen receptor-expressing cell lines [44]. Nevertheless, the phenolic and flavonoid-rich crude ethanol extracts of PF-L exhibited the lowest required concentration to become 50% cytotoxic to DU-145 cells. It indicates that PF-L has deficient activity relative to potent anticancer plant extracts [45,46,47]. This activity could signify that metabolites at low concentrations are more bioactive or that major metabolites require high concentrations. Deciphering the probable molecular targets of these metabolites are explored with network pharmacology and molecular docking analyses. This network-based bioinformatic analysis of metabolite targets and PRAD revealed 60 affected targets. The results show that compounds **1**, **5**, **15**, **23**, **24**, **32**, **36**, and **37** have the most relevant targets on PCa cytotoxicity.

Previous studies revealed that many of the main compounds and such derivatives have been able to impact PCa cell proliferation and growth. It was known that apigenin targeted PI3K/Akt/Fox-O, β-catenin, and insulin-like growth factor-I signaling pathways, impacting PCa proliferation [48]. Also, apigenin derivatives have been known to induce cell death and cell cycle arrest [49]. Coumaric acid is known to have a similar effect, but it has targeted mitochondrial-related apoptosis, downregulated specific cyclin-dependent kinases, and decreased the expression of specific oncogenes [50,51]. Perillaldehyde has been shown to reduce bone metastasis of PC-3 prostate cancer cells with repression of the nuclear factor-κB (NF-κB) pathway and receptor activator of NF-κB ligand (RANKL) [52]. At the same time, scutellarin is considered a promising cancer treatment due to its multifactorial effect of cell cycle arrest, apoptosis induction, and angiogenic and metastatic reduction [53,54,55,56]. However, there are limited studies regarding their structure analogs with PCa. The major compound of PF-L, RA, was observed to have an excellent activity to DU-145, which could be the main-effect compound of its extracts [57,58,59,60].

In this study, affected targets from PRAD with PF-L were subjected to a GO term, KEGG, and Reactome pathway enrichment analysis, which uncovered prospective cytotoxic mechanisms to PCa in silico. Relevant processes that could be involved in this phenomenon from GO enrichment are the response to oxygen-containing compounds, protein phosphorylation, cellular component disassembly, and to a lesser extent, angiogenesis. From pathway enrichment, PF-L metabolites potentially regulate the AMPK signaling pathway, EGFR tyrosine kinase inhibitor resistance, miRNAs in cancer, prostate cancer, steroid and lipid metabolism, SCF-KIT and PIK3/Akt signaling pathways, EPH-ephrin mediated cell repulsion, and the VEGFA-VEGFR2 pathway. Collectively, these pathways were discovered to significantly affect the tumorigenesis, growth, progression, energy regulation, metastasis, and adhesion of PCa cells [61,62,63,64,65,66,67,68,69].

One of the relevant protein-coding genes from the PPI network is Fyn, under the Src family kinases. This gene is typically overexpressed in PCa, a signaling protein typically attributed to tumor cell proliferation and metastasis [70,71]. It regulates diverse biological functions, such as cell growth, proliferation, migration, and adhesion. However, overexpressed Fyn mediates the PIK3/Akt pathway to induce anti-apoptotic mechanisms and EMT for cell invasion and metastatic initiation, preventing the cell death pathways of normal cells [70,71,72]. It is a recent interest for cancer targets due to their functions in cancer cells and their drug resistance ability; however, the latter is effective in direct gene expression regulation. Indeed, previously reported immunochemical assays for Fyn kinase activity of RA and caffeic acid exhibited noncompetitive inhibition [73,74,75].

The molecular action of PF-L metabolites was also implicated with ITGB3, a transmembrane integrin receptor primarily responsible for cell adhesion, migration, and macrophage phagocytosis ability [76,77]. It was previously reported that the antagonism of integrin α_V_β_3_ or vitronectin receptor revealed the most effective reduction in tumor cell angiogenesis and metastasis compared to the other classes of integrins [76,77,78,79]. Since it is overexpressed in highly metastatic neuroendocrinal metastasis of PCa, it is an antiangiogenic target, and it affects the migration, survival, plasticity, and metastatic ability of tumor cells as signal initiators [76,78]. However, previous results for endometrial cells showed that exposure to aqueous extracts of PF-S&L corresponded to increased integrin β3 expression and no observable changes in integrin αV [80]. This result is unfavorable for cancer angiogenesis and should be considered for further study.

Another PPI network-significant protein-coding gene with PF-L is the PIK3R1 from the PIK3/Akt pathway, which typically regulates cell death, proliferation, metabolism, and angiogenesis [81]. It is an obligate heterodimer composed of the p110α catalytic subunit (PIK3CA) and the p85α regulatory subunit (PIK3R1) [82]. This protein-coding gene is underexpressed and altered in both primary and metastatic PCa cells and has inverse negative feedback with androgen receptor (AR) [81,83]. Hence, the dynamic interplay of PIK3R1 and AR indicates that the current androgen-deprivation treatment could enable cell proliferation and survivability, suggesting vital consideration of combinatorial inhibition of the two targets [81]. For PF-L, the PI3K/Akt/NF-κB axis was regarded as the primary pathway of inhibited migration and invasion, induced apoptosis, and cell cycle arrest from RA [75,84,85].

Lastly, PF-L metabolites were shown to have significant interaction with PDGFRs, which are transmembrane receptor tyrosine kinases that can initiate various pathways with the SH2 domain-containing signal transduction molecules as inducers of cell growth and division, such as in the PI3K/Akt pathway [86]. Dimerization of these receptor monomers with PDGF ligands induces autophosphorylation [86,87,88]. The PDGFRα and β were implicated in PCa due to their heightened expression in PCa cells and oncogenic activity on its growth, angiogenesis, and metastatic potential [86,89,90,91]. In fact, ligand and dimerization-free PDGFRα are about as bone-metastatic compared to the complete form of the receptor for PCa, indicative of PDGF-independent mechanism on its metastatic ability [89,90]. In comparison, the high expression of PDGFRβ in prostate tumor stroma was associated with PCa aggressiveness and low patient survivability [92,93]. PDGF-induced proliferation of mesangial cells was inhibited upon exposure to RA [94].

Molecular docking was utilized to test whether the selected metabolites could interact with the key targets. This simulation revealed stable binding with respective proteins consistent with previous activities of their analogs. RA had the best activity, which could affirm the activity of PF-L on PCa. These results suggest that added structural features in the main compound may have increased their potency.

Anti-PCa activity from PF-L extracts can be promising, especially as a preventative measure and an additional diet for individuals with a high risk of PCa development. However, pharmacokinetics and toxicity with normal cells in vitro and in vivo tests are required to successfully evaluate PF-L’s potential as an anticancer agent for PCa or other cancer types. Also, the polar and nonpolar metabolites present in PF-L could be synergistic or antagonistic for the anti-PCa activity of PF and could be addressed from compound isolation and comparative explorations. At the same time, predicted mechanisms and actions from bioinformatic analyses should be further validated and investigated with gene expression studies. Meta-analysis of PCa patients and people at risk with their diet on such TCMs could provide an expansive vantage point of its potential as a cancer curative or preventative agent. Interestingly, other environmental conditions for the plant may lead to unique secondary metabolites, hence different activity.

## 4. Materials and Methods

### 4.1. Preparation of Plant Extracts

Dried *P. frutescens* stem (PF-S), leaves (PF-L), and seeds (PF-SD) were acquired from a local TCM store located in Tainan City, Taiwan. The identification of *P. frutescens* was authenticated by Dr. Chia-Jung Lee, Ph.D. Program in Clinical Drug Development of Herbal Medicine, College of Pharmacy, Taipei Medical University. A voucher specimen was deposited as #2023-CJCU-PF-001 at the Department of Medical Sciences Industry at Chang Jung Christian University, Taiwan.

The samples were collected from manually separated leaves and stems, where the petioles were included with the latter. These samples were mechanically ground for surface area enlargement and particle size reduction for effective extraction. For ethanol extracts, dried samples (50 g) were immersed with one-liter 95% ethanol under a two-hour 65 °C water bath and total reflux. For water extracts, dried samples (50 g) were leached with one liter of distilled and deionized water in a traditional Chinese decoction pot until reduced to ~200 mL. The crude extracts were vacuum-filtered with 0.45 µm filter paper, concentrated by vacuum evaporation, and lyophilized to obtain a solvent-free extract stored in a −20 °C refrigerator for further analysis. The yield from the extraction is reported in Table 2.

### 4.2. Total Phytochemical Content Assay

The total phytochemical contents of PF extracts were assessed according to Tsai et al. [95] with slight modifications. A 1000 μg/mL stock solution of crude PF extracts was prepared for all assays. The chemicals used in the analyses are reagent grade. Standards were prepared from two-fold serial dilutions of 1000 μg/mL stock solution from 500 to 15.60 μg/mL. Results of the following assays were expressed in milligrams of standard equivalents per gram of crude extract. All measurements were taken in triplicates and color-corrected with blank solutions.

#### 4.2.1. Total Phenolics Content (TPC) Assay

Gallic acid standards were prepared for TPC analysis. The standard and sample solutions (80 μL) were added with 400 μL of 0.2 N Folin–Ciocâlteu reagent. After five-minute equilibration, 320 μL of 7.5% (*w*/*v*) Na_2_CO_3_ was added. The mixture was incubated for 30 min at room temperature and transferred to a 96-well microarray plate. The absorbance of the mixtures was measured at 600 nm.

#### 4.2.2. Total Flavonoids Content (TFC) Assay

Rutin standards were prepared for TFC analysis. The standard and sample solutions (500 μL) were reacted with 500 μL of 2.0% (*w*/*v*) AlCl_3_ reagent. The mixture was incubated for 15 min at room temperature and transferred to a 96-well microarray plate. The absorbance of the mixtures was measured at 430 nm.

### 4.3. Anti-Prostate Cancer Activity

#### 4.3.1. Human Prostate Cancer Cell Line DU-145

The cell culture of DU-145 was adapted from Park et al. [96]. Briefly, human prostate cancer cell line DU-145 was obtained from Bioresource Collection and Research Center (BCRC, Taiwan). The DU-145 cells were cultured in Eagle’s Minimum Essential Medium (EMEM), containing 10% fetal bovine serum (FBS) and 1% penicillin-streptomycin. The cultured cells were kept at 37 °C in a humidified atmosphere containing 5% CO_2_. The cells were sub-cultured within two-day intervals after reaching 70–80% confluence.

#### 4.3.2. Cell Treatment and Cell Viability with WST-1 Assay

DU-145 cells were cultured in 96-well microarray plates (2 × 10^4^ cells/well) and incubated at 37 °C. After 24 h of incubation, the cells were treated with various concentrations (two-fold dilution from 5.00 to 0.625 mg/mL) of crude extracts for another 24 h. The positive control in this analysis was 1.00 mg/mL of 5-fluorouracil (5FU). Cell viability was determined using a WST-1 assay (Abcam^TM^) [97]. Briefly, 100 µL of a fresh medium was placed in each treated well with DU-145 cells. A total of 10 µL of WST-1 reagent was added to each well. The plate was incubated for 2 h at 37 °C. The absorbances were measured at 570 nm with background control. Measurements were performed in triplicates for statistical significance and reproducibility.

### 4.4. Chromatographic Analysis

The major compound was separated and identified in the extracts with liquid chromatography (LC-2050C 3D; Shimadzu Corporation, Japan) for verification through a Hypersil™ BDS C18 column (250 × 4.6 mm, 5 µm, Thermo Fischer Scientific Inc., Taiwan) kept at 25 °C. Chromatography conditions were adapted from Lee et al. [30] with slight modifications. A 5 μL of 10 mg/mL crude extracts dissolved in methanol was filtered with a 0.45 µm syringe filter and injected into the column. Chromatography runs were performed with mobile phases of 0.05% trifluoroacetic acid in water (eluent A) and methanol (eluent B). The implemented gradient program was 30% B (0–5 min); 30-50% B (5–20 min); 50–0% B (20-40 min); 90–100% B (40–45 min); and 100% B (45–50 min); then, it was equilibrated back to initial conditions for five minutes at a flow rate of 0.4 mL/min. Data acquisition was set until 45 min.

### 4.5. Data Treatment and Statistical Analysis

Microsoft Excel was used for data processing, and GraphPad Prism software was used for statistical analysis and data visualization. Experimental data are reported as mean ± standard deviation (SD). Multiple comparisons of means were conducted through one-way ANOVA and Dunnett’s pairwise comparison test. A statistical significance of *p* < 0.05 was set throughout the analysis.

### 4.6. Network Pharmacology

A brief prediction of the mechanism of action of PF with PCa cytotoxicity was performed with network analysis adapted from Jin et al. [98] and Peng et al. [99] with modifications. The methods and resulting data of this analysis were validated according to the guidelines of the World Federation of Chinese Medicine Societies [100].

#### 4.6.1. Target Prediction and Identification

The experimental and predicted gene targets of previously identified putative metabolites from PF extracts were identified from SuperPred (prediction.charite.de, accessed in June 2023). This target prediction tool utilizes logistic regression and 2048-long Morgan fingerprints with 94.1% target prediction accuracy [101]. The default set cut-offs for predicted target probability and model accuracy were ≥ 50%. Differentially expressed genes between PRAD (ICD-11: 2C82.0) and normal prostate cells were determined from the Gene Expression Profiling Interactive Analysis (GEPIA2, gepia2.cancer-pku.cn, accessed in June 2023). Genomic datasets of the GEPIA2 web server utilized were from TCGA and GTEx isoform expression data [102]. Comparisons were performed with the LIMMA differential method. Statistically significant genes were selected with the set significance of *p* < 0.01 and |log_2_ FC|> 1. Gene nomenclatures were standardized into their official gene symbols using SynGO (www.syngoportal.org/convert, accessed in June 2023) [103]. Matched gene targets between the metabolites and PRAD were selected for further analysis with Venny 2.1 (bioinfogp.cnb.csic.es/tools/venny/, accessed in June 2023) and Intervene (asntech.shinyapps.io/intervene, accessed in June 2023) [104,105].

#### 4.6.2. Network Construction

The identified matched protein-coding genes were imported to the STRING database 11.5 (string-db.org, accessed in June 2023) for the construction of a PPI network [106,107,108,109,110,111,112,113,114,115,116,117,118]. Furthermore, proteins with more than one (1) interaction and FDR stringency of 0.01 limited to the “*Homo sapiens*” species were considered significant. The PPI network is transferred CytoScape 3.9.1 for further analysis [119]. Protein relevance in the network was evaluated with topological analysis using MCC and DMNC algorithms from the cytoHubba plug-in in CytoScape [34]. The metabolite target network was constructed similarly and analyzed with the degree-ranking algorithm.

#### 4.6.3. Enrichment Analysis

Functional profiling was performed with g:Profiler (biit.cs.ut.ee/gprofiler/gost, accessed in June 2023) for the exploration of the predicted pharmacological mechanism and signaling pathways involved in the action of metabolites to the selected targets [120,121]. Relevant results from databases of gene ontology (GO) terms [122,123], KEGG [124,125,126] and Reactome [127,128,129,130,131,132,133,134] pathways were characterized from a g:SCS significance threshold of less than 0.05 limited to the “*Homo sapiens*” species. Secondary data filtering of GO terms was automatically performed from g:Profiler with a simple greedy search algorithm. Data visualizations were performed in Python 3.11.

#### 4.6.4. Molecular Docking Validation

The molecular modeling and visualization software BIOVIA Discovery Studio was employed for the docking analysis. Three-dimensional (3D) structure-data files (SDFs) of ligand molecules were collected from PubChem (chem.ncbi.nlm.nih.gov, accessed in June 2023). The ligands were prepared with the ligand preparation (at pH 7.5 ± 1.0) and minimization protocols in Discovery Studio. Experimentally elucidated structures of protein targets were collected in PDB file format from the Protein Data Bank (PDB, www.rcsb.org, accessed in June 2023). Afterward, water molecules and irrelevant heteroatoms were removed, and polar hydrogens were added to the protein structure. The proteins were prepared with the protein preparation protocol (at pH 7.4 and 0.145 M ionic strength) in Discovery Studio. These proteins were minimized until an energy gradient of 0.01 with CHARMm force field and the Momany-Rone partial charge estimation method. The defined binding sites were determined from the co-crystallized ligands volume or eraser algorithm in Discovery Studio [135]. The docking runs were performed using the grid-based CDOCKER protocol [136]. Receptor-ligand binding site visualizations were performed in PyMOL™ [137].

## 5. Conclusions

Most PF extracts follow dose–response behavior for PCa cell cytotoxicity with DU-145 cells, where its ethanolic leaf extracts had the most cytotoxic activity. While characterized as having minimal activity as an anti-prostate cancer agent, the plant may have preventative potential for PCa development and progression. Regardless, bioinformatic analyses revealed that many minor metabolites and the major metabolite, RA, could have contributed to the cytotoxic potential of the mentioned extract, affecting the determined key targets FYN, PDGFRA, PDGFRB, PIK3R1, and ITGB3. Molecular docking of the compounds to the key targets consistently verified the protein–ligand interactions from network pharmacology results. Hence, the cytotoxicity of the leaf extract can be postulated from cell death induction and cell cycle arrest from action to the key targets. More advanced studies, such as in vivo trials, gene expression, or clinical trials, are recommended to verify activities and mechanisms of action from PF-L and their metabolites to PCa.

## Figures and Tables

**Table 1:** Total phytochemical content of *P. frutescens* extracts.

Extract	TPC (mg GAE/g CE)	TFC (mg RE/g CE)
PF-S-E	32.1602 ± 0.5445	18.8969 ± 0.9848
PF-S-W	30.1641 ± 1.0134	N.D.
PF-L-E	83.1263 ± 0.9431	42.6199 ± 1.9120
PF-L-W	87.2611 ± 1.0841	54.3026 ± 1.9616
PF-SD-E	22.2391 ± 0.2058	27.3490 ± 0.8821
PF-SD-W	44.6299 ± 0.4715	11.6966 ± 0.3097
*Calibration Curve*	y = 5.6109x − 0.0192 R^2^ = 0.9990	y = 5.5923x + 0.0280 R^2^ = 0.9994

Abbreviations: TPC, total phenolics content; TFC, total flavonoids content; GAE, gallic acid equivalents; CE, crude extract; RE, rutin equivalents; S, stem; L, leaf; SD, seed; E, ethanol extract; W, water extract; N.D., not detected.

**Table 2:** Extraction yield (g CE/100 g PM) of lyophilized *P. frutescens* crude extracts.

	Plant Material		
Solvent	*Stem*	*Leaf*	*Seed*
Ethanol	1.8856	6.5310	3.2080
Water	4.9060	15.5686	4.0512

Abbreviations: CE, crude extract; PM, dried plant material.

## Data Availability

The data and results presented in this study are available on request from the first author and corresponding author.

## Supplementary Materials

The following supporting information can be downloaded at: https://www.mdpi.com/article/10.3390/plants12163006/s1, Figure S1: Metabolite-target network of *P. frutescens* leaves against PRAD; Figure S2: Protein-protein interaction network of intersected genes set; Figure S3: Metabolite-target network of PIN-relevant proteins; Figure S4: GO term enrichment analysis by (**a**) molecular function, (**b**) biological process, and (**c**) cellular component; (**d**) KEGG and Reactome pathway enrichment analysis; Figure S5: Affected proteins in the AMPK signaling pathway from the KEGG database (has04152); Figure S6: Affected proteins in the EGFR tyrosine kinase inhibitor resistance pathway from the KEGG database (hsa01521); Figure S7: Affected proteins from protein-translated miRNAs in cancer from the KEGG database (hsa05206); Figure S8: Affected proteins in the prostate cancer pathway from the KEGG database (hsa05215); Figure S9: Affected proteins in the steroid metabolism pathway from the Reactome database (R-HSA-8957322); Figure S10: Affected proteins in the SCF-KIT signaling pathway from the Reactome database (R-HSA-1433557); Figure S11: Affected proteins in the lipid metabolism pathway from the Reactome database (R-HAS-556833); Figure S12: Affected proteins in the PI3K/Akt signaling pathway in cancers from the Reactome database (R-HAS-2219528); Figure S13: Affected proteins in the EPH-ephrin mediated cell repulsion pathway from the Reactome database (R-HSA-2682334); Figure S14: Affected proteins in the VEGFA-VEGFR2 pathway from the Reactome database (R-HAS-194138); Table S1: Summary of putative compounds of *P. frutescens* leaf extracts from metabolomic studies; Table S2: First-pass relevant gene ontology (GO) terms; Table S3: Second-pass relevant gene ontology (GO) terms; Table S4: Identified protein targets from protein-protein interaction network analysis; and, Table S5: CDOCKER energy (in kcal/mol) of protein-ligand complexes.
