# Supplementary material for: An In Vitro Evaluation and Network Pharmacology Analysis of Prospective Anti-Prostate Cancer Activity from Perilla frutescens
Source: Plants (Basel). 2023 Aug 21;12(16):3006. doi: 10.3390/plants12163006 (PMC10457999; doi:10.3390/plants12163006)
Supplement: Supplementary file 1 [file plants-12-03006-s001.zip › plants-2551860-supplementary.pdf]

Supplementary Material for "An *in vitro* evaluation and network pharmacology analysis of prospective anti-prostate cancer activity from *Perilla frutescens*."

Table S1. Summary of putative compounds of *P. frutescens* leaf extracts from metabolomic studies.

Index	Name	SMILES	Ref.
**1**[*,**]	5′-Gluco-pyranosyoxyjasmanic acid	OCC1OC(OCC\C=C\CC2C(CC(=O)O)CCC2=O)C(O)C(O)C1O	[1–3]
**2**	Acetyloxycaffeic acid	CC(=O)O\C(=C/c1ccc(O)c(O)c1)\C(=O)O	[3]
**3**	Apigenin	Oc1ccc(cc1)C2=CC(=O)c3c(O)cc(O)cc3O2	[1,3]
**4**	Apigenin-7-*o*-diglucoside	OCC1OC(O[C@@]2(Oc3cc(O)c4C(=O)C=C(Oc4c3)c5ccc(O)cc5)OC(CO)C(O)C(O)C2O)C(O)C(O)C1O	[3]
**5**[**]	Apigenin-7-*o*-diglucuronide	O[C@H]1[C@H](O)[C@@H](O[C@H]2[C@H](O)[C@@H](O)[C@@H](O[C@@H]2Oc3cc(O)c4C(=O)C=C(Oc4c3)c5ccc(O)cc5)C(=O)O)O[C@H]([C@@H]1O)C(=O)O	[1,3]
**6**	Apigenin-7-*o*-glucoside	OC[C@H]1O[C@@H](Oc2cc(O)c3C(=O)C=C(Oc3c2)c4ccc(O)cc4)[C@H](O)[C@@H](O)[C@@H]1O	[3]
**7**	Apigenin-7-*o*-glucuronide	O[C@H]1[C@H](Oc2cc(O)c3C(=O)C=C(Oc3c2)c4ccc(O)cc4)O[C@@H]([C@@H](O)[C@@H]1O)C(=O)O	[1,3]
**8**	Caffeic acid	OC(=O)\C=C\c1ccc(O)c(O)c1	[1]
**9**	Caffeic acid 3-*o*-glucuronide	O[C@@H]1[C@@H](O)[C@H](Oc2cc(\C=C\C(=O)O)ccc2O)O[C@@H]([C@H]1O)C(=O)O	[3]
**10**	Caffeic acid ethyl ester	CCOC(=O)\C=C\c1ccc(O)c(O)c1	[3]
**11**	Caffeic acid tetramer	COC(=O)C(Cc1ccc(O)c(O)c1)OC(=O)C2=Cc3cc(O)c(O)cc3C(C2C(=O)OC(Cc4ccc(O)c(O)c4)C(=O)O)c5ccc(O)c(O)c5	[3,4]
**12**	Chrysoeriol	COc1cc(ccc1O)C2=CC(=O)c3c(O)cc(O)cc3O2	[1,3]
**13**	Cis-shisonin	[Cl-].OC[C@H]1O[C@@H](Oc2cc(O)cc3[o+]c(c(O[C@@H]4O[C@H](COC(=O)\C=C/c5ccc(O)cc5)[C@@H](O)[C@H](O)[C@H]4O)cc23)c6ccc(O)c(O)c6)[C@H](O)[C@@H](O)[C@@H]1O	[3]
**14**	Corosolic acid	C[C@@H]1CC[C@@]2(CC[C@]3(C)C(=CC[C@@H]4[C@@]5(C)C[C@@H](O)[C@H](O)C(C)(C)[C@@H]5CC[C@@]34C)[C@@H]2[C@H]1C)C(=O)O	[1]
**15**[**]	Coumaric acid-4-*o*-glucoside	OC[C@H]1O[C@@H](Oc2ccc(\C=C\C(=O)O)cc2)[C@H](O)[C@@H](O)[C@@H]1O	[3]
**16**	Liquiritigenin	Oc1ccc(cc1)[C@@H]2CC(=O)c3ccc(O)cc3O2	[1]
**17**	Loganin	COC(=O)C1=CO[C@@H](O[C@@H]2O[C@H](CO)[C@@H](O)[C@H](O)[C@H]2O)[C@@H]3[C@@H](C)[C@@H](O)C[C@H]13	[1]
**18**	Luteolin	Oc1cc(O)c2C(=O)C=C(Oc2c1)c3ccc(O)c(O)c3	[3]
**19**	Luteolin-7-*o*-diglucuronide	O[C@@H]1[C@@H](O)[C@H](OC(=O)[C@H]2O[C@@H](Oc3cc(O)c4C(=O)C=C(Oc4c3)c5ccc(O)c(O)c5)[C@H](O)[C@@H](O)[C@@H]2O)O[C@@H]([C@H]1O)C(=O)O	[3]
**20**	Luteolin-7-*o*-glucuronide	O[C@@H]1[C@@H](O)[C@H](Oc2cc(O)c3C(=O)C=C(Oc3c2)c4ccc(O)c(O)c4)O[C@@H]([C@H]1O)C(=O)O	[3]
**21**	Luteoloside	OC[C@H]1O[C@@H](Oc2cc(O)c3C(=O)C=C(Oc3c2)c4ccc(O)c(O)c4)[C@H](O)[C@@H](O)[C@@H]1O	[3]
**22**	Malonyl-shisonin	O[C@H]1[C@H](O)[C@@H](COC(=O)CC(=O)O)O[C@@H](Oc2cc(O)cc3[o+]c(c(O[C@@H]4O[C@H](COC(=O)\C=C\c5ccc(O)cc5)[C@@H](O)[C@@H](O)[C@H]4O)cc23)c6ccc(O)c(O)c6)[C@@H]1O	[3]

**23***	*n*-Octanoylsucrose	CCCCCCCC(=O)OC[C@H]1O[C@@](CO)(O[C@H]2O[C@H](CO)[C@@H](O)[C@H](O)[C@H]2O)[C@@H](O)[C@@H]1O	[1]
**24****	Perillaldehyde	CC(=C)C1CCC(=CC1)C=O	[5]
**25**	Protocatechuic acid-3-*o*-glucoside	OCC1OC(Oc2cc(ccc2O)C(=O)O)C(O)C(O)C1O	[3]
**26**	Quercetin-3-*o*-glucoside	OC[C@H]1O[C@@H](OC2=C(Oc3cc(O)cc(O)c3C2=O)c4ccc(O)c(O)c4)[C@H](O)[C@@H](O)[C@@H]1O	[3]
**27**	Quercetin-3-*o*-glucuronide	O[C@@H]1[C@@H](O)[C@H](OC2=C(Oc3cc(O)cc(O)c3C2=O)c4ccc(O)c(O)c4)O[C@@H]([C@H]1O)C(=O)O	[3]
**28#**	Rosmarinic acid	OC(=O)[C@@H](Cc1ccc(O)c(O)c1)OC(=O)\C=C\c2ccc(O)c(O)c2	[1,3]
**29**	Rosmarinic acid methyl ester	COC(=O)[C@@H](Cc1ccc(O)c(O)c1)OC(=O)\C=C\c2ccc(O)c(O)c2	[1,3]
**30**	Rosmarinic acid-3-*o*-glucoside	OC[C@@H]1O[C@H](Oc2cc(C[C@@H](OC(=O)\C=C\c3ccc(O)c(O)c3)C(=O)[O-])ccc2O)[C@@H](O)[C@H](O)[C@H]1O	[1]
**31**	Sagerinic acid	OC(=O)[C@@H](Cc1ccc(O)c(O)c1)OC(=O)[C@H]2[C@H]([C@H]([C@H]2c3ccc(O)c(O)c3)c4ccc(O)c(O)c4)C(=O)O[C@H](Cc5ccc(O)c(O)c5)C(=O)O	[1]
**32***	Scutellarein-7-*o*-glucuronide	O[C@@H]1[C@@H](O)[C@H](Oc2cc3OC(=CC(=O)c3c(O)c2O)c4ccc(O)cc4)O[C@@H]([C@H]1O)C(=O)O	[1,3]
**33**	Scutellarin-7-*o*-diglucuronide	O[C@@H]1[C@@H](O)[C@H](Oc2cc3OC(=CC(=O)c3c(O)c2O[C@@H]4O[C@@H]([C@@H](O)[C@H](O)[C@H]4O)C(=O)O)c5ccc(O)cc5)O[C@@H]([C@H]1O)C(=O)O	[3]
**34**	Shisonin	OC[C@H]1O[C@@H](Oc2cc(O)cc3[o+]c(c(O[C@H]4O[C@H](COC(=O)\C=C\c5ccc(O)cc5)[C@H](O)[C@H](O)[C@H]4O)cc23)c6ccc(O)c(O)c6)[C@H](O)[C@@H](O)[C@@H]1O	[3]
**35**	Tomentic acid	C[C@@H]1CC[C@@]2(CC[C@]3(C)C(=CC[C@@H]4[C@@]5(C)C[C@@H](O)[C@H](O)C(C)(C)[C@@H]5CC[C@@]34C)[C@@H]2[C@]1(C)O)C(=O)O	[1]
**36****	trans-*p*-Menth-8-en-yl caffeate	CC1CCC(C(C1)OC(=O)\C=C\c2ccc(O)c(O)c2)C(=C)C	[1]
**37*,****	Tuberonic acid	OCC\C=C/C[C@H]1[C@@H](CC(=O)O)CCC1=O	[3]

* key metabolite from metabolite-target network; ** protein-protein interaction network (PIN)-relevant metabolites; # major metabolite.

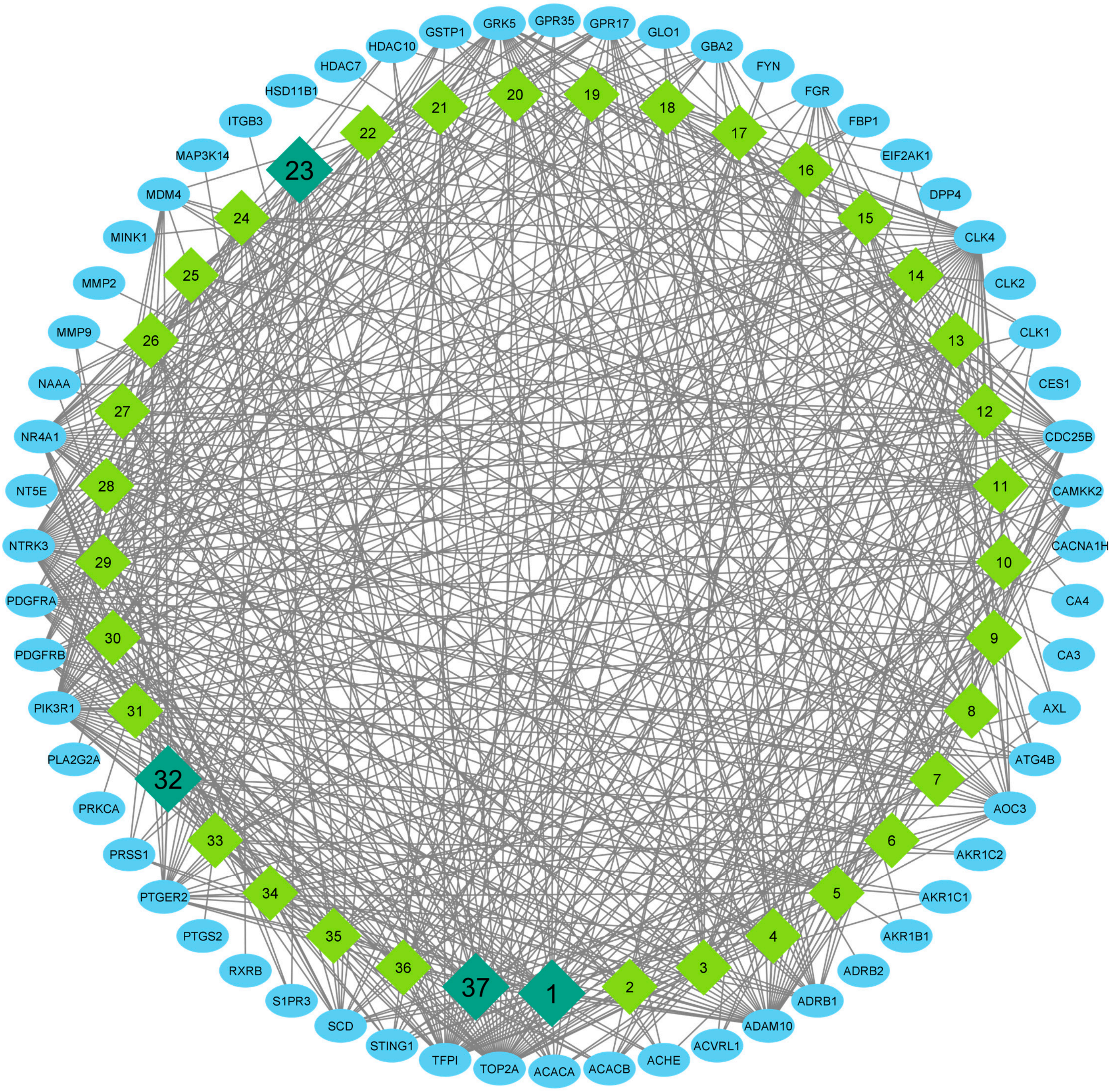

Figure S1. Metabolite-target network of *P. frutescens* leaves against PRAD.

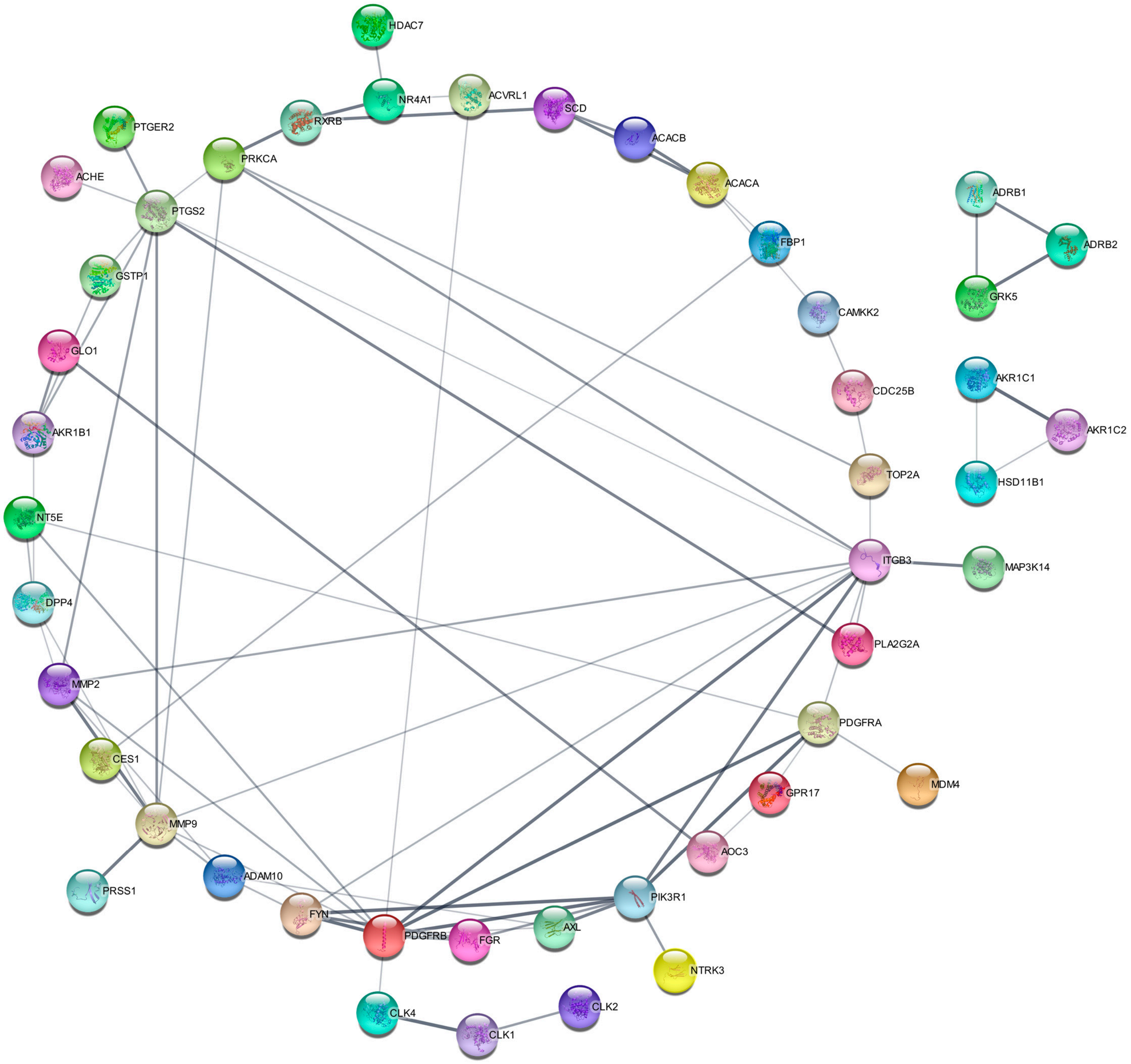

Figure S2. Protein-protein interaction network of intersected genes set where thicker lines indicate high interaction.

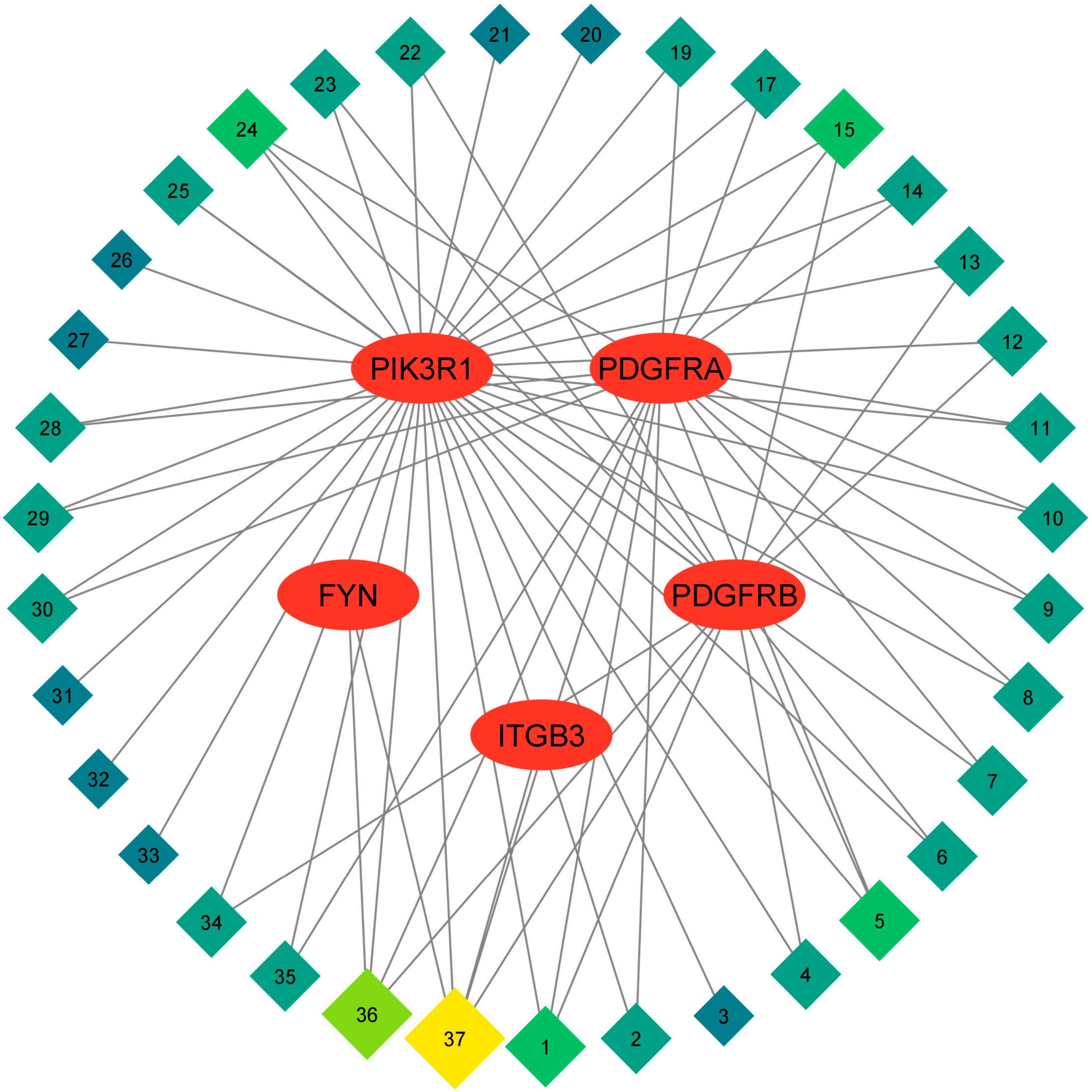

Figure S3. Metabolite-target network of PIN-relevant proteins.

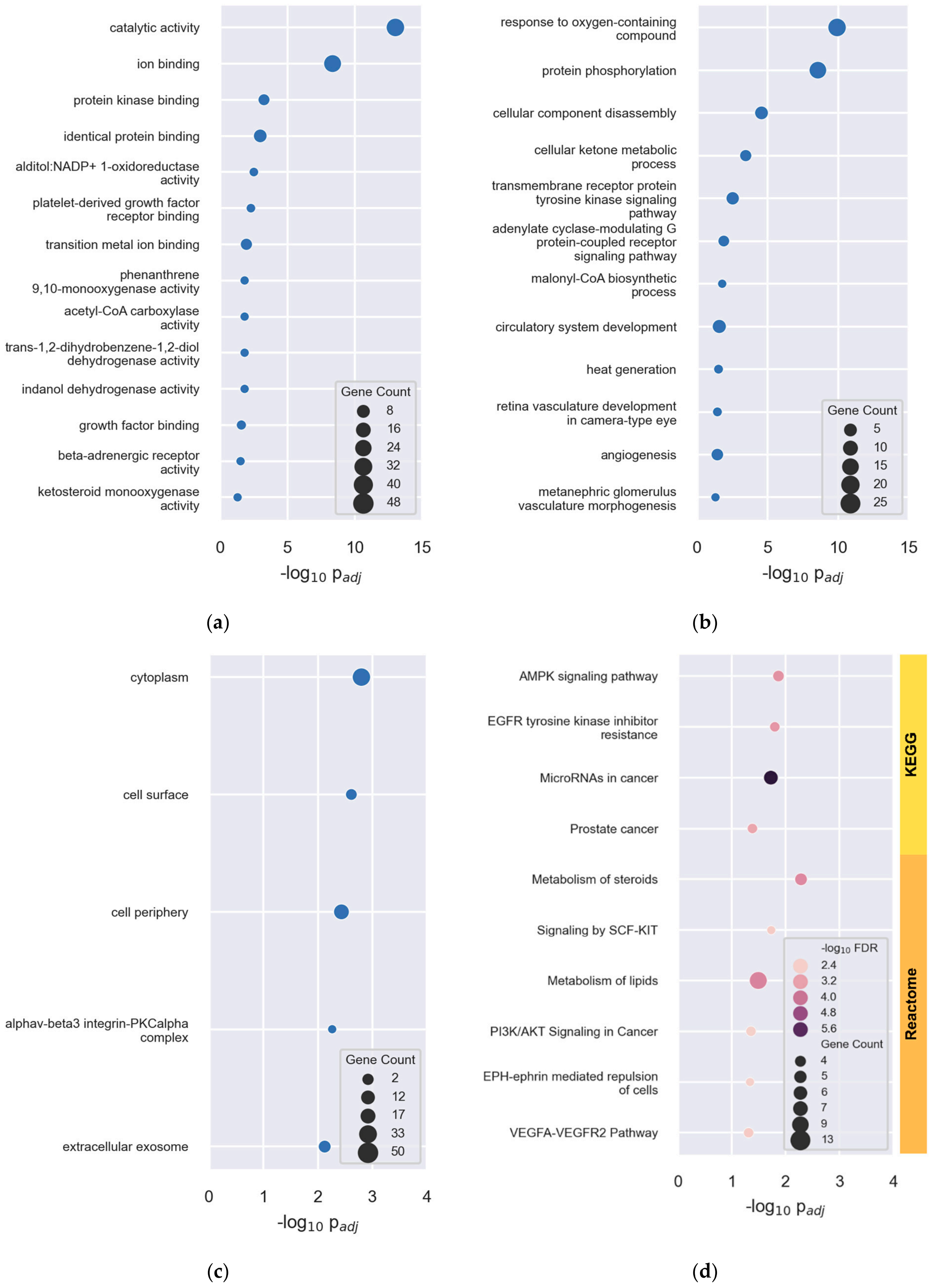

Figure S4. GO term enrichment analysis by (**a**) molecular function, (**b**) biological process, and (**c**) cellular component; (**d**) KEGG and Reactome pathway enrichment analysis.

Table S2. First-pass relevant gene ontology (GO) terms.

GO Term ID	Name	$-\log_{10} p_{adj}$	I.S.
Molecular function			
GO:0004672	protein kinase activity	8.2535	16
GO:0004713	protein tyrosine kinase activity	8.0118	10
GO:0016301	kinase activity	7.2990	17
GO:0016773	phosphotransferase activity, alcohol group as acceptor	7.1555	16
GO:0140096	catalytic activity, acting on a protein	6.4744	26
GO:0030554	adenyl nucleotide binding	6.3544	22
GO:0032559	adenyl ribonucleotide binding	6.0344	21
GO:0016772	transferase activity, transferring phosphorus-containing groups	6.0022	17
GO:0036094	small molecule binding	5.5785	26
GO:0000166	nucleotide binding	4.8919	23
GO:1901265	nucleoside phosphate binding	4.8882	23
GO:0017076	purine nucleotide binding	4.7731	22
GO:0005524	ATP binding	4.7360	19
GO:0043168	anion binding	4.5967	24
GO:0032555	purine ribonucleotide binding	4.4429	21
GO:0032553	ribonucleotide binding	4.3768	21
GO:0004674	protein serine/threonine kinase activity	3.4843	10
GO:0035639	purine ribonucleoside triphosphate binding	3.2951	19
GO:0097367	carbohydrate derivative binding	3.1077	21
GO:0019900	kinase binding	2.7616	12
GO:0046983	protein dimerization activity	2.7584	14
GO:0019199	transmembrane receptor protein kinase activity	2.5901	5
GO:0019899	enzyme binding	2.4865	19
GO:0016740	transferase activity	2.3707	20
GO:0106310	protein serine kinase activity	2.2088	8
GO:0043169	cation binding	2.0098	28
GO:0005080	protein kinase C binding	1.9094	4
GO:0005017	platelet-derived growth factor receptor activity	1.8257	2
GO:0008270	zinc ion binding	1.7447	11
GO:0004714	transmembrane receptor protein tyrosine kinase activity	1.6749	4
GO:0042803	protein homodimerization activity	1.6211	10
GO:0008106	alcohol dehydrogenase (NADP+) activity	1.5569	3
GO:0038085	vascular endothelial growth factor binding	1.5255	2
Biological process			
GO:0070887	cellular response to chemical stimulus	9.8061	32
GO:0010033	response to organic substance	9.4219	32
GO:1901701	cellular response to oxygen-containing compound	9.2854	23
GO:0042221	response to chemical	8.6955	37
GO:0006796	phosphate-containing compound metabolic process	7.9048	30
GO:0006793	phosphorus metabolic process	7.8039	30
GO:0006950	response to stress	7.4382	35
GO:0016310	phosphorylation	7.2284	24
GO:0046777	protein autophosphorylation	7.0241	11
GO:0071310	cellular response to organic substance	6.9545	25
GO:0051239	regulation of multicellular organismal process	6.9126	30
GO:0009605	response to external stimulus	6.5721	29

GO Term ID	Name	$-log_{10}$ p_{adj}	I.S.
GO:0050896	response to stimulus	6.5203	50
GO:0033993	response to lipid	6.0852	17
GO:0030154	cell differentiation	5.7925	34
GO:0048869	cellular developmental process	5.7203	34
GO:0065008	regulation of biological quality	5.5878	28
GO:1901564	organonitrogen compound metabolic process	5.4415	41
GO:0071495	cellular response to endogenous stimulus	5.4333	20
GO:0032101	regulation of response to external stimulus	5.2667	17
GO:0051716	cellular response to stimulus	5.2179	44
GO:0009967	positive regulation of signal transduction	5.0897	20
GO:0009719	response to endogenous stimulus	5.0476	21
GO:0023056	positive regulation of signaling	4.9491	21
GO:0048518	positive regulation of biological process	4.9410	40
GO:0010647	positive regulation of cell communication	4.9402	21
GO:0008284	positive regulation of cell population proliferation	4.8862	16
GO:0018108	peptidyl-tyrosine phosphorylation	4.8392	11
GO:0018212	peptidyl-tyrosine modification	4.8151	11
GO:0008283	cell population proliferation	4.6625	22
GO:0032502	developmental process	4.6599	40
GO:0071396	cellular response to lipid	4.6416	13
GO:0009628	response to abiotic stimulus	4.6184	17
GO:0048583	regulation of response to stimulus	4.5528	31
GO:0009725	response to hormone	4.5023	15
GO:0042127	regulation of cell population proliferation	4.4284	20
GO:0048584	positive regulation of response to stimulus	4.3086	23
GO:0030335	positive regulation of cell migration	4.2497	12
GO:1902531	regulation of intracellular signal transduction	4.2315	20
GO:0030334	regulation of cell migration	4.1700	15
GO:0006954	inflammatory response	4.0742	14
GO:0006629	lipid metabolic process	4.0503	18
GO:0009966	regulation of signal transduction	4.0479	26
GO:2000147	positive regulation of cell motility	4.0226	12
GO:0048856	anatomical structure development	3.9864	37
GO:0048522	positive regulation of cellular process	3.9594	36
GO:0048519	negative regulation of biological process	3.9109	37
GO:0040017	positive regulation of locomotion	3.8887	12
GO:0000302	response to reactive oxygen species	3.8070	8
GO:2000145	regulation of cell motility	3.8066	15
GO:1902533	positive regulation of intracellular signal transduction	3.7552	15
GO:0060548	negative regulation of cell death	3.7100	15
GO:0019538	protein metabolic process	3.6963	35
GO:0016477	cell migration	3.6576	18
GO:0042325	regulation of phosphorylation	3.6003	16
GO:0040012	regulation of locomotion	3.5550	15
GO:0023051	regulation of signaling	3.5430	27
GO:0032501	multicellular organismal process	3.5304	42
GO:0010646	regulation of cell communication	3.5162	27
GO:0040011	locomotion	3.5112	17

GO Term ID	Name	-log$_{10}$ p$_{adj}$	I.S.
GO:0032787	monocarboxylic acid metabolic process	3.4660	12
GO:0006631	fatty acid metabolic process	3.4536	10
GO:0048523	negative regulation of cellular process	3.3875	32
GO:0042327	positive regulation of phosphorylation	3.3170	13
GO:0006692	prostanoid metabolic process	3.2482	5
GO:0006693	prostaglandin metabolic process	3.2482	5
GO:0016137	glycoside metabolic process	3.2402	4
GO:0006979	response to oxidative stress	3.2010	10
GO:1902644	tertiary alcohol metabolic process	3.0632	4
GO:0008152	metabolic process	3.0587	57
GO:0050790	regulation of catalytic activity	3.0587	19
GO:0035556	intracellular signal transduction	3.0554	23
GO:0062197	cellular response to chemical stress	3.0297	9
GO:0036211	protein modification process	3.0172	27
GO:0044283	small molecule biosynthetic process	2.9187	11
GO:0097305	response to alcohol	2.9129	8
GO:0019220	regulation of phosphate metabolic process	2.8899	16
GO:0051174	regulation of phosphorus metabolic process	2.8855	16
GO:0048870	cell motility	2.8517	18
GO:0045937	positive regulation of phosphate metabolic process	2.8483	13
GO:0010562	positive regulation of phosphorus metabolic process	2.8483	13
GO:0044255	cellular lipid metabolic process	2.8481	14
GO:0033559	unsaturated fatty acid metabolic process	2.7932	6
GO:0022617	extracellular matrix disassembly	2.7654	5
GO:0032870	cellular response to hormone stimulus	2.7023	11
GO:0050727	regulation of inflammatory response	2.6639	9
GO:0065009	regulation of molecular function	2.6562	22
GO:0018193	peptidyl-amino acid modification	2.6514	15
GO:0048871	multicellular organismal-level homeostasis	2.5629	12
GO:0043412	macromolecule modification	2.4871	27
GO:0044597	daunorubicin metabolic process	2.4232	3
GO:0051241	negative regulation of multicellular organismal process	2.3725	14
GO:0010941	regulation of cell death	2.3636	17
GO:0010631	epithelial cell migration	2.3497	8
GO:0090132	epithelium migration	2.3178	8
GO:0044281	small molecule metabolic process	2.2879	18
GO:0042592	homeostatic process	2.2742	17
GO:0050794	regulation of cellular process	2.2738	49
GO:0030647	aminoglycoside antibiotic metabolic process	2.2692	3
GO:0030638	polyketide metabolic process	2.2692	3
GO:0044598	doxorubicin metabolic process	2.2692	3
GO:0090130	tissue migration	2.2551	8
GO:1901698	response to nitrogen compound	2.2548	14
GO:0043542	endothelial cell migration	2.2534	7
GO:0065007	biological regulation	2.2316	53
GO:0034614	cellular response to reactive oxygen species	2.1907	6
GO:0051240	positive regulation of multicellular organismal process	2.1657	17
GO:0050789	regulation of biological process	2.1602	52

GO Term ID	Name	-log$_{10}$ p$_{adj}$	I.S.
GO:0008202	steroid metabolic process	2.1534	8
GO:1901657	glycosyl compound metabolic process	2.1501	5
GO:1901699	cellular response to nitrogen compound	2.0788	11
GO:0033674	positive regulation of kinase activity	2.0772	9
GO:0008207	C21-steroid hormone metabolic process	2.0740	4
GO:0048661	positive regulation of smooth muscle cell proliferation	2.0466	5
GO:0007165	signal transduction	1.9956	34
GO:0043549	regulation of kinase activity	1.9627	11
GO:0104004	cellular response to environmental stimulus	1.9510	8
GO:0071214	cellular response to abiotic stimulus	1.9510	8
GO:0042445	hormone metabolic process	1.9180	7
GO:0043066	negative regulation of apoptotic process	1.9097	12
GO:0120254	olefinic compound metabolic process	1.9072	6
GO:0031323	regulation of cellular metabolic process	1.9048	32
GO:0019222	regulation of metabolic process	1.9015	38
GO:0007275	multicellular organism development	1.8799	29
GO:0010243	response to organonitrogen compound	1.8457	13
GO:0048513	animal organ development	1.8451	23
GO:0009888	tissue development	1.8343	18
GO:0044238	primary metabolic process	1.8093	52
GO:0043069	negative regulation of programmed cell death	1.8063	12
GO:0001932	regulation of protein phosphorylation	1.7930	13
GO:0043551	regulation of phosphatidylinositol 3-kinase activity	1.7754	4
GO:0080134	regulation of response to stress	1.7554	15
GO:0044237	cellular metabolic process	1.7470	49
GO:0097306	cellular response to alcohol	1.7428	5
GO:0048771	tissue remodeling	1.7261	6
GO:1904645	response to amyloid-beta	1.6991	4
GO:0000165	MAPK cascade	1.6936	11
GO:0014070	response to organic cyclic compound	1.6634	12
GO:1901652	response to peptide	1.5903	9
GO:0019752	carboxylic acid metabolic process	1.5895	12
GO:0007154	cell communication	1.5562	35
GO:0034308	primary alcohol metabolic process	1.5370	5
GO:0048013	ephrin receptor signaling pathway	1.5225	4
GO:0043436	oxoacid metabolic process	1.4903	12
GO:0071417	cellular response to organonitrogen compound	1.4844	10
GO:0006082	organic acid metabolic process	1.4637	12
GO:0032102	negative regulation of response to external stimulus	1.4492	8
GO:0071704	organic substance metabolic process	1.4306	54
GO:0007566	embryo implantation	1.4249	4
GO:0007167	enzyme-linked receptor protein signaling pathway	1.4155	12
GO:0009056	catabolic process	1.4129	20
GO:0051347	positive regulation of transferase activity	1.4053	9
GO:0043534	blood vessel endothelial cell migration	1.4047	5
GO:0009893	positive regulation of metabolic process	1.3692	25
GO:0001503	ossification	1.3423	8

GO Term ID	Name	-log$_{10}$ p$_{adj}$	I.S.
GO:0038091	positive regulation of cell proliferation by VEGF-activated platelet derived growth factor receptor signaling pathway	1.3049	2
GO:0072277	metanephric glomerular capillary formation	1.3049	2
GO:0072275	metanephric glomerulus morphogenesis	1.3049	2
GO:0038086	VEGF-activated platelet-derived growth factor receptor signaling pathway	1.3049	2
GO:0043550	regulation of lipid kinase activity	1.3033	4
Cellular component			
GO:0097038	perinuclear endoplasmic reticulum	2.2109	3
GO:0005886	plasma membrane	2.2100	31
GO:1903561	extracellular vesicle	2.0615	17
GO:0043230	extracellular organelle	2.0588	17
GO:0065010	extracellular membrane-bounded organelle	2.0588	17
GO:0012505	endomembrane system	1.9609	27
GO:0031982	vesicle	1.8395	24
GO:0005783	endoplasmic reticulum	1.6701	16
GO:0005615	extracellular space	1.4791	21
GO:0030141	secretory granule	1.4781	10

Abbreviations: I.S., intersected set of genes.

Table S3. Second-pass relevant gene ontology (GO) terms.

GO Term ID	Name	-log$_{10}$ p$_{adj}$	I.S.
Molecular function			
GO:0003824	catalytic activity	13.0079	48
GO:0043167	ion binding	8.3496	44
GO:0019901	protein kinase binding	3.2617	12
GO:0042802	identical protein binding	2.9848	20
GO:0004032	alditol:NADP+ 1-oxidoreductase activity	2.5096	3
GO:0005161	platelet-derived growth factor receptor binding	2.2928	3
GO:0046914	transition metal ion binding	1.9572	13
GO:0018636	phenanthrene 9,10-monooxygenase activity	1.8257	2
GO:0003989	acetyl-CoA carboxylase activity	1.8257	2
GO:0047115	trans-1,2-dihydrobenzene-1,2-diol dehydrogenase activity	1.8257	2
GO:0047718	indanol dehydrogenase activity	1.8257	2
GO:0019838	growth factor binding	1.5848	5
GO:0004939	beta-adrenergic receptor activity	1.5255	2
GO:0047086	ketosteroid monooxygenase activity	1.3045	2
Biological process			
GO:1901700	response to oxygen-containing compound	9.9174	27
GO:0006468	protein phosphorylation	8.5583	24
GO:0022411	cellular component disassembly	4.5661	12
GO:0042180	cellular ketone metabolic process	3.4483	8
GO:0007169	transmembrane receptor protein tyrosine kinase signaling pathway	2.5244	11
GO:0007188	adenylate cyclase-modulating G protein-coupled receptor signaling pathway	1.8948	7
GO:2001295	malonyl-CoA biosynthetic process	1.7812	2
GO:0072359	circulatory system development	1.5766	13
GO:0031649	heat generation	1.5220	3

GO Term ID	Name	$-log_{10}\ p_{adj}$	I.S.
GO:0061298	retina vasculature development in camera-type eye	1.4437	3
GO:0001525	angiogenesis	1.4375	9
GO:0072276	metanephric glomerulus vasculature morphogenesis	1.3049	2
Cellular component			
GO:0005737	cytoplasm	2.7986	50
GO:0009986	cell surface	2.6140	12
GO:0071944	cell periphery	2.4320	33
GO:0035866	alphav-beta3 integrin-PKCalpha complex	2.2620	2
GO:0070062	extracellular exosome	2.1246	17

Abbreviations: I.S., intersected set of genes.

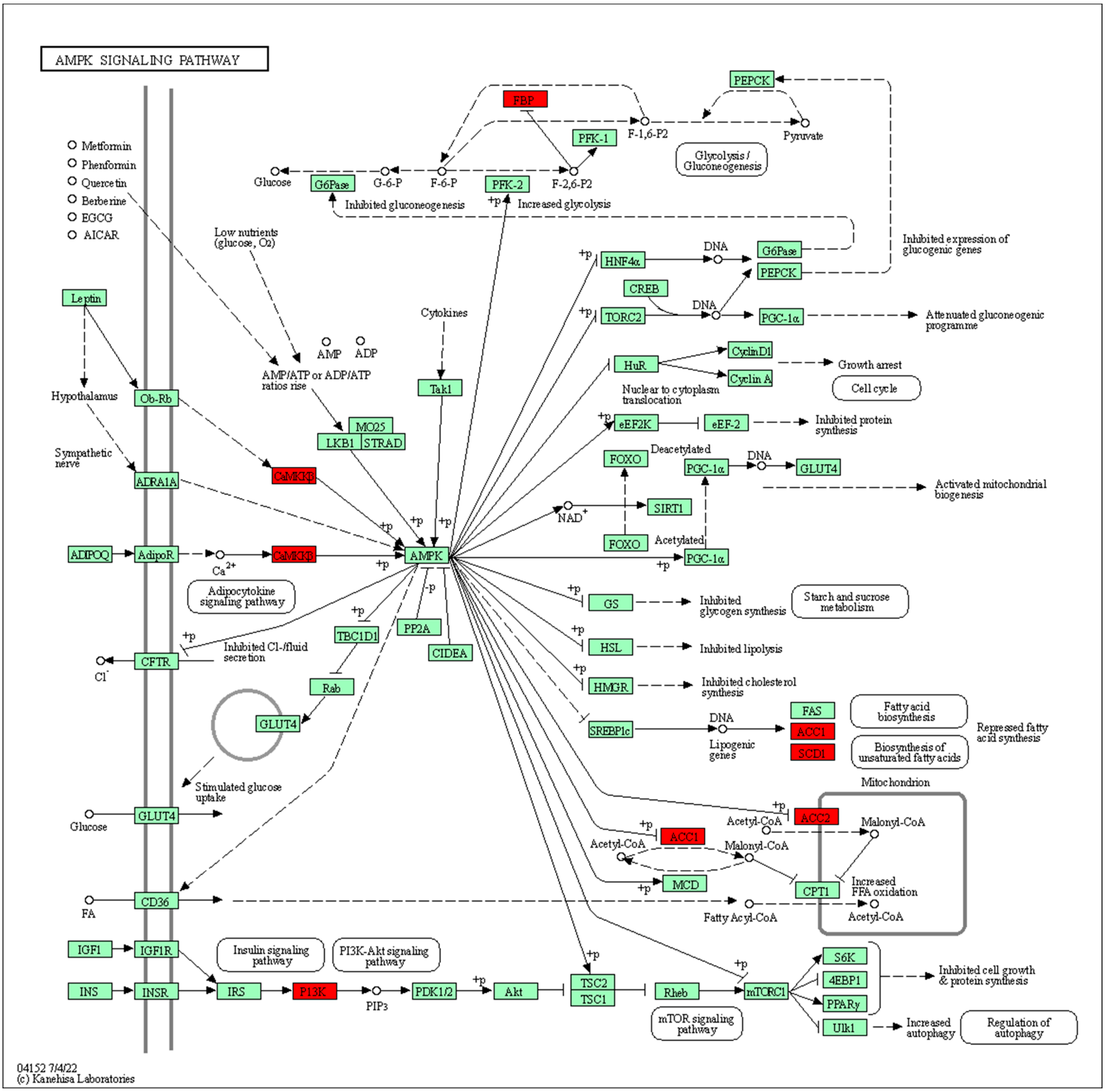

Figure S5. Affected proteins in the AMPK signaling pathway from the KEGG database (hsa04152).

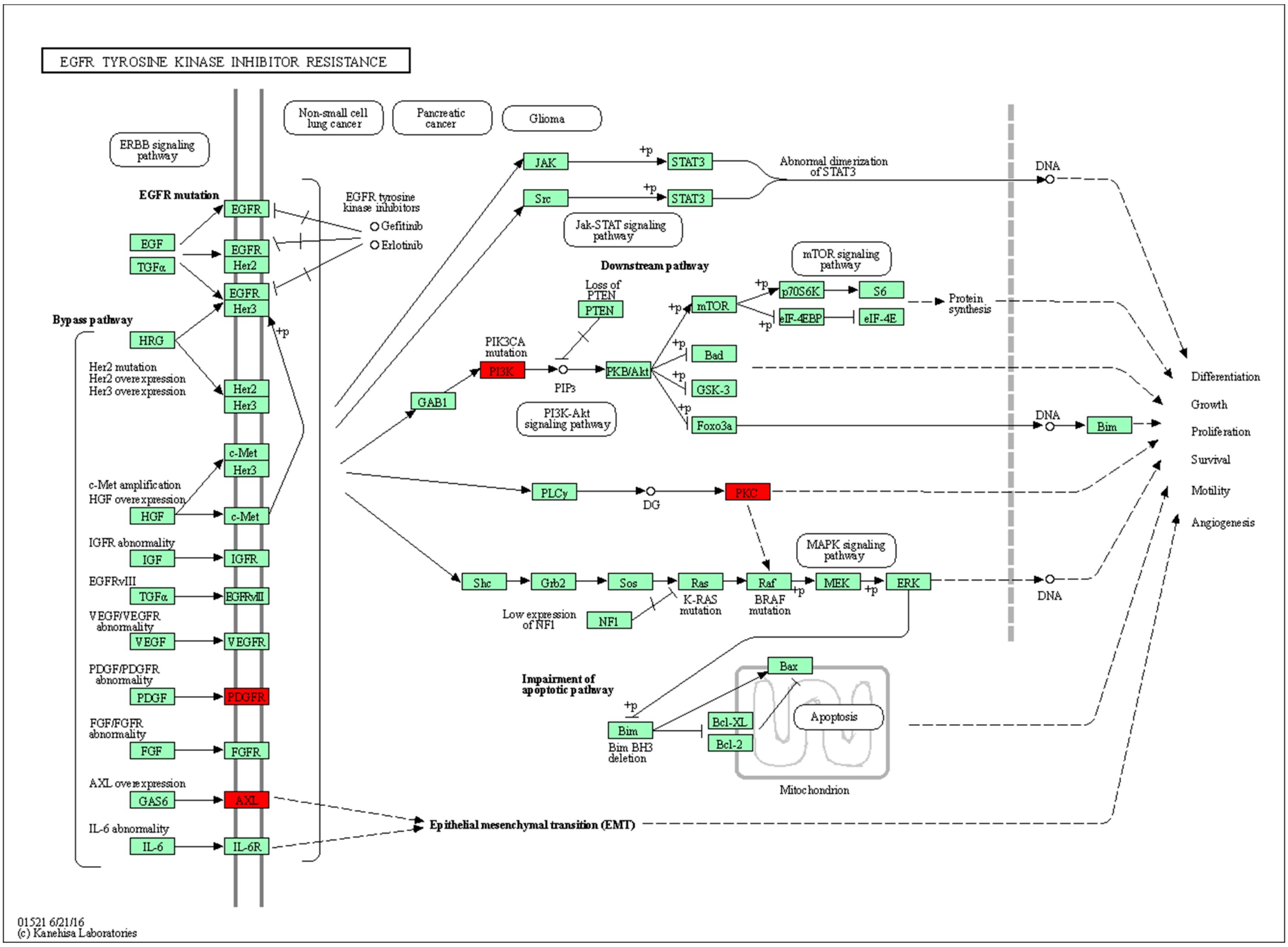

Figure S6. Affected proteins in the EGFR tyrosine kinase inhibitor resistance pathway from the KEGG database (hsa01521).

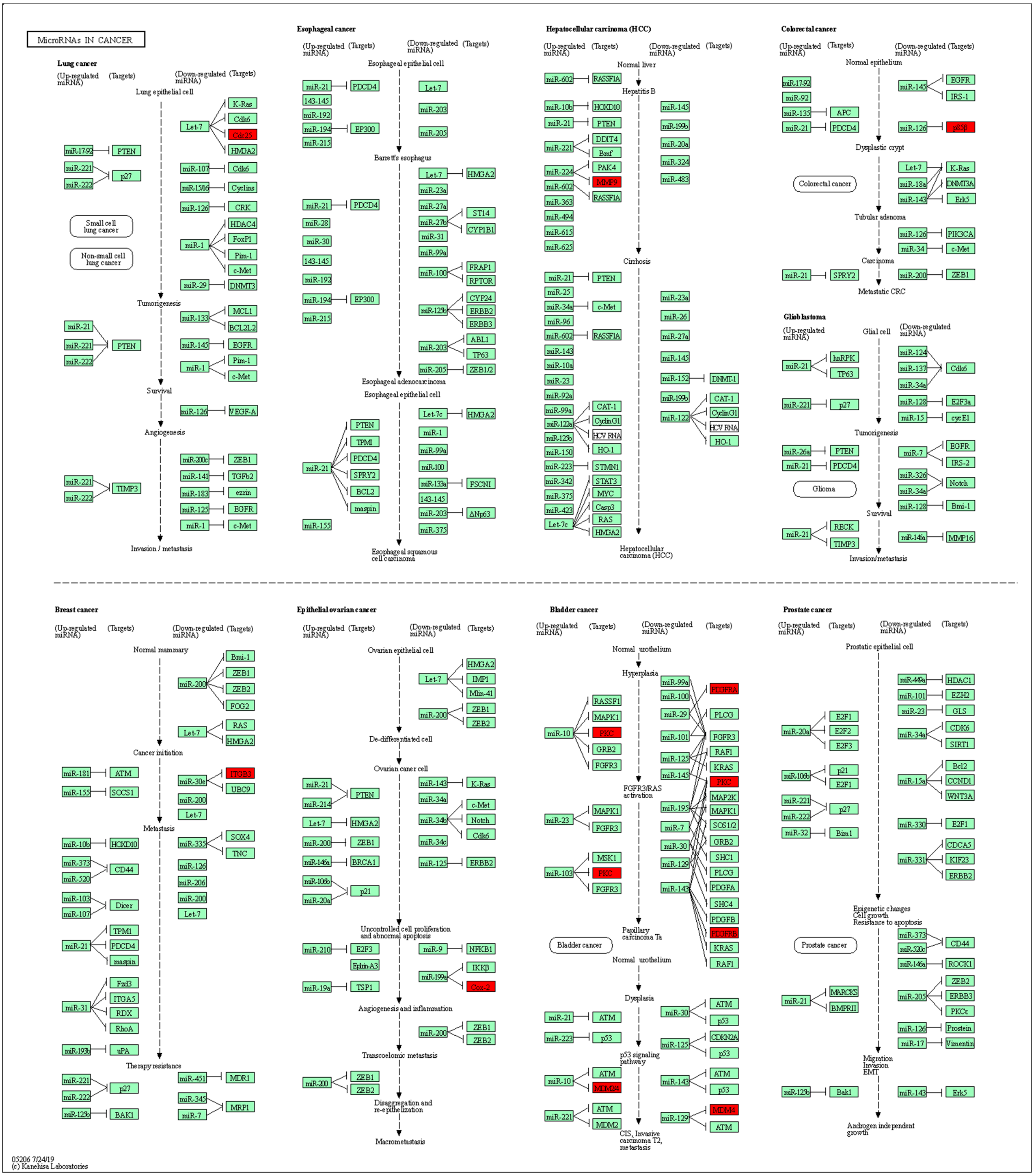

Figure S7. Affected proteins from protein-translated miRNAs in cancer from the KEGG database (hsa05206).

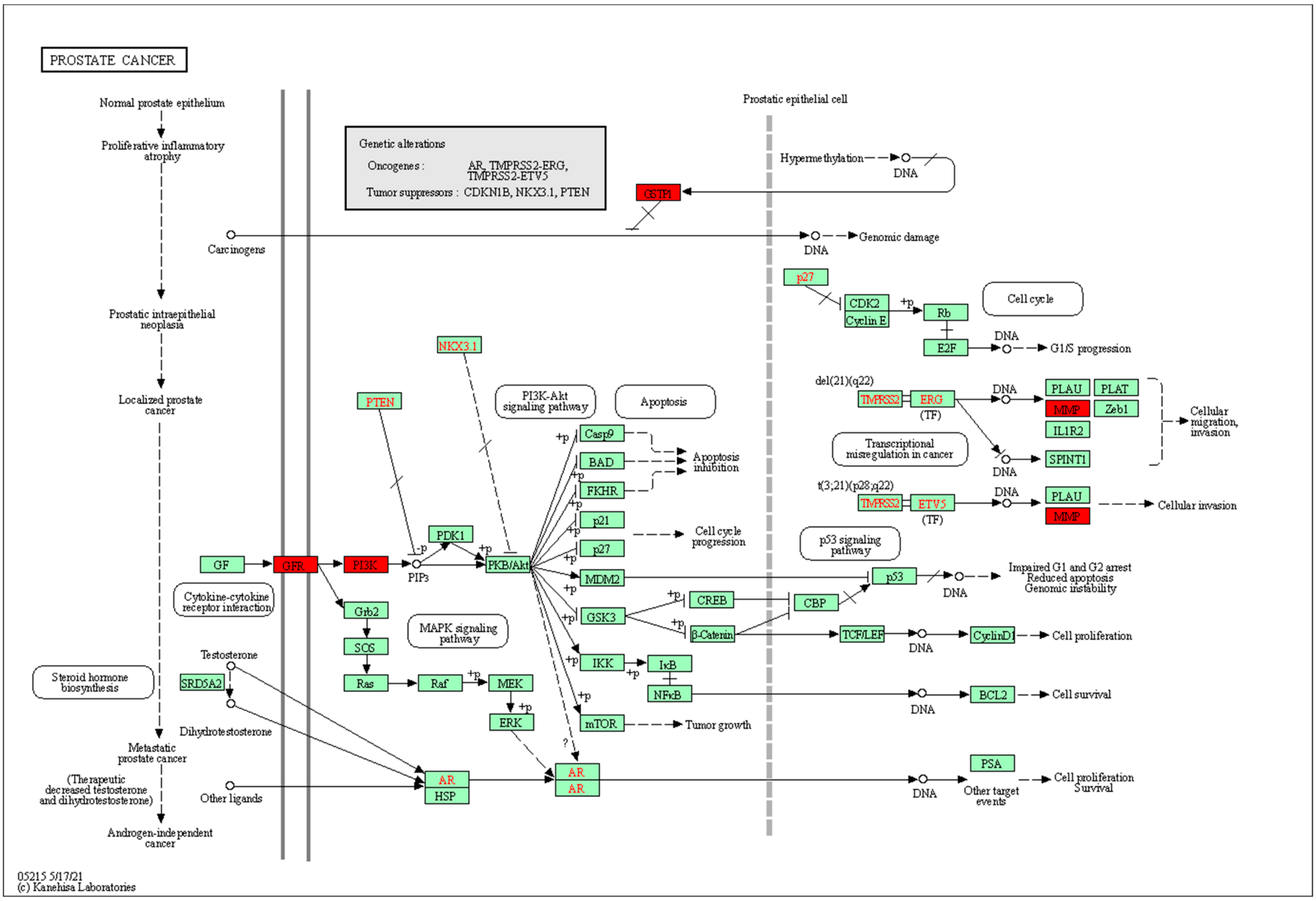

Figure S8. Affected proteins in the prostate cancer pathway from the KEGG database (hsa05215).

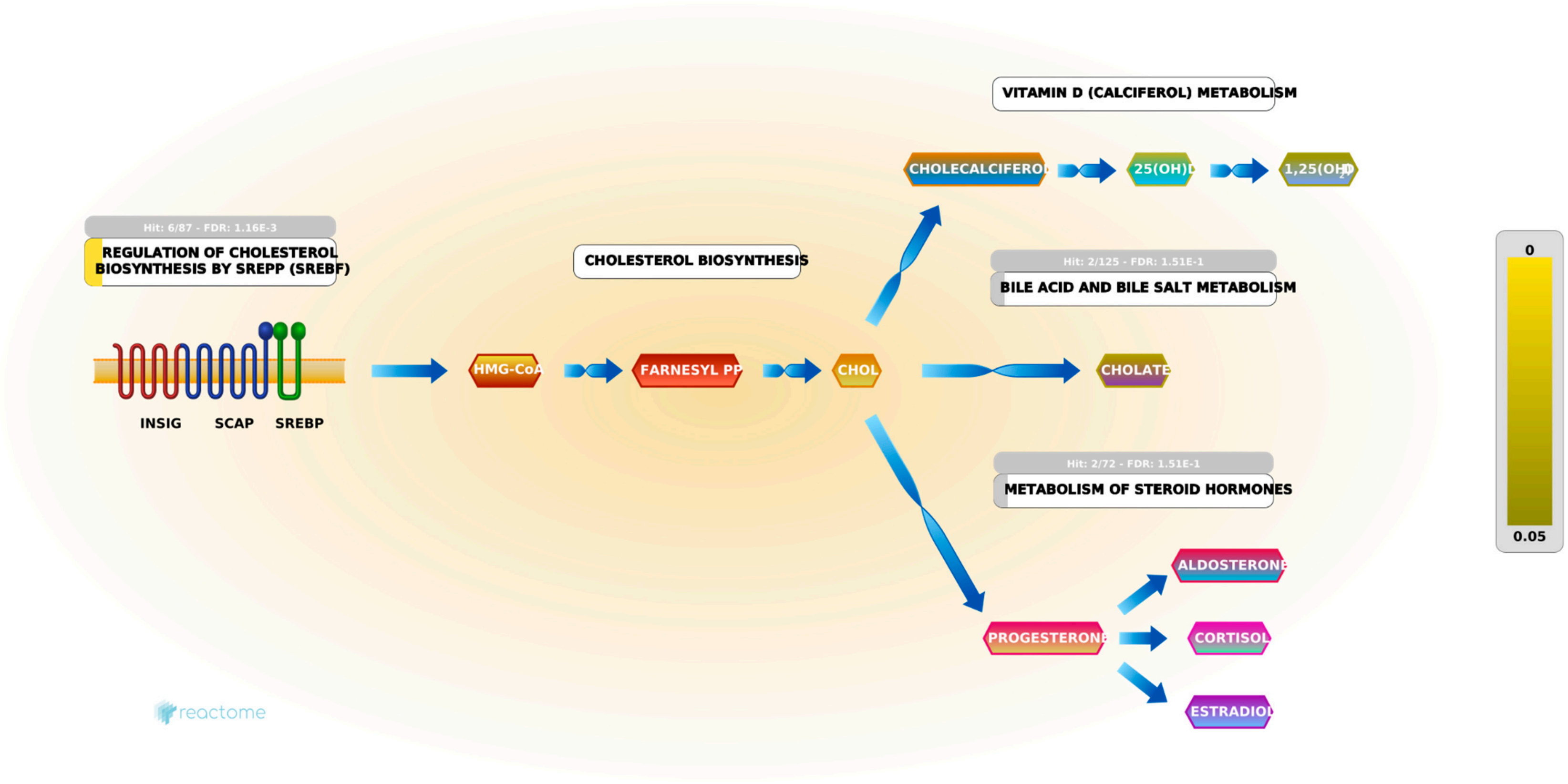

Figure S9. Affected proteins in the steroid metabolism pathway from the Reactome database (R-HSA-8957322).

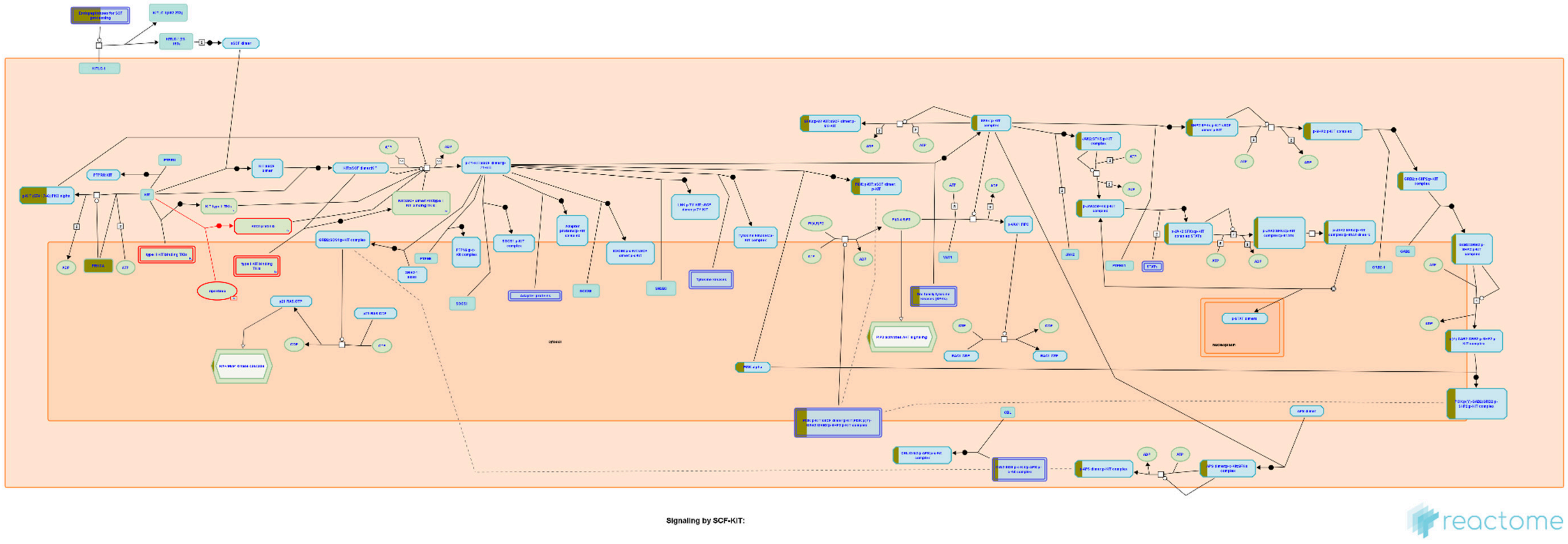

Figure S10. Affected proteins in the SCF-KIT signaling pathway from the Reactome database (R-HSA-1433557).

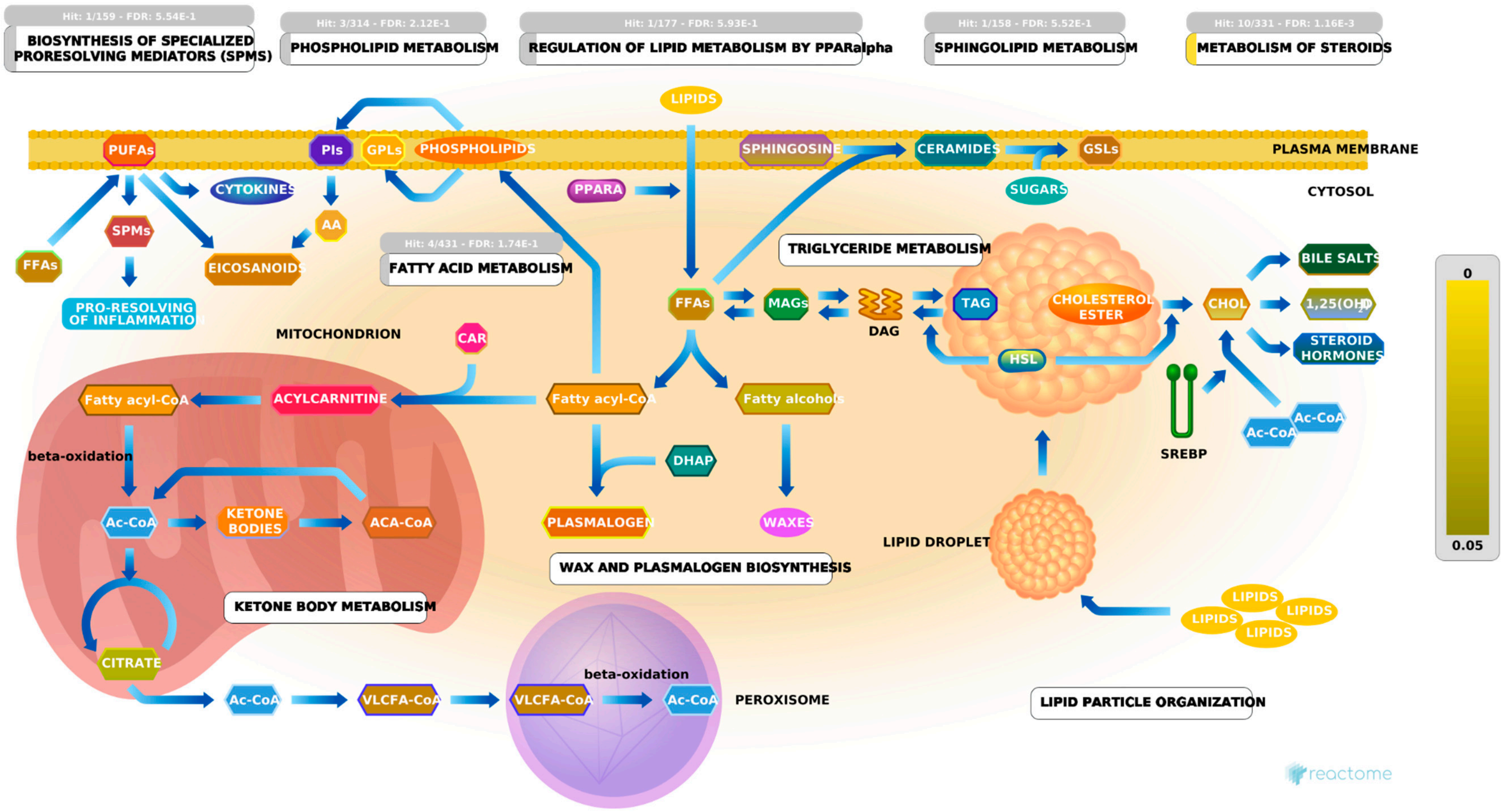

Figure S11. Affected proteins in the lipid metabolism pathway from the Reactome database (R-HSA-556833).

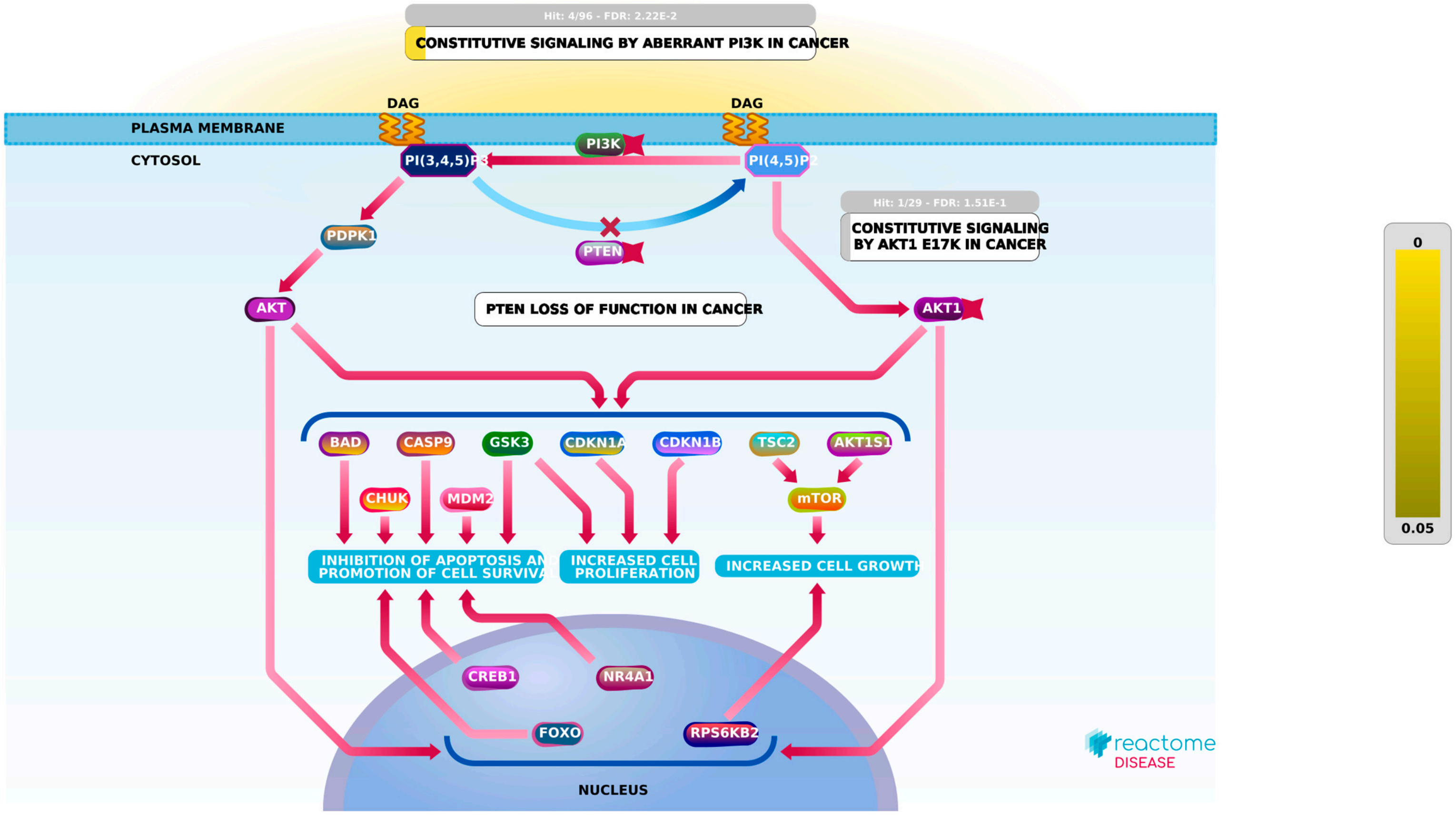

Figure S12. Affected proteins in the PI3K/Akt signaling pathway in cancers from the Reactome database (R-HSA-2219528).

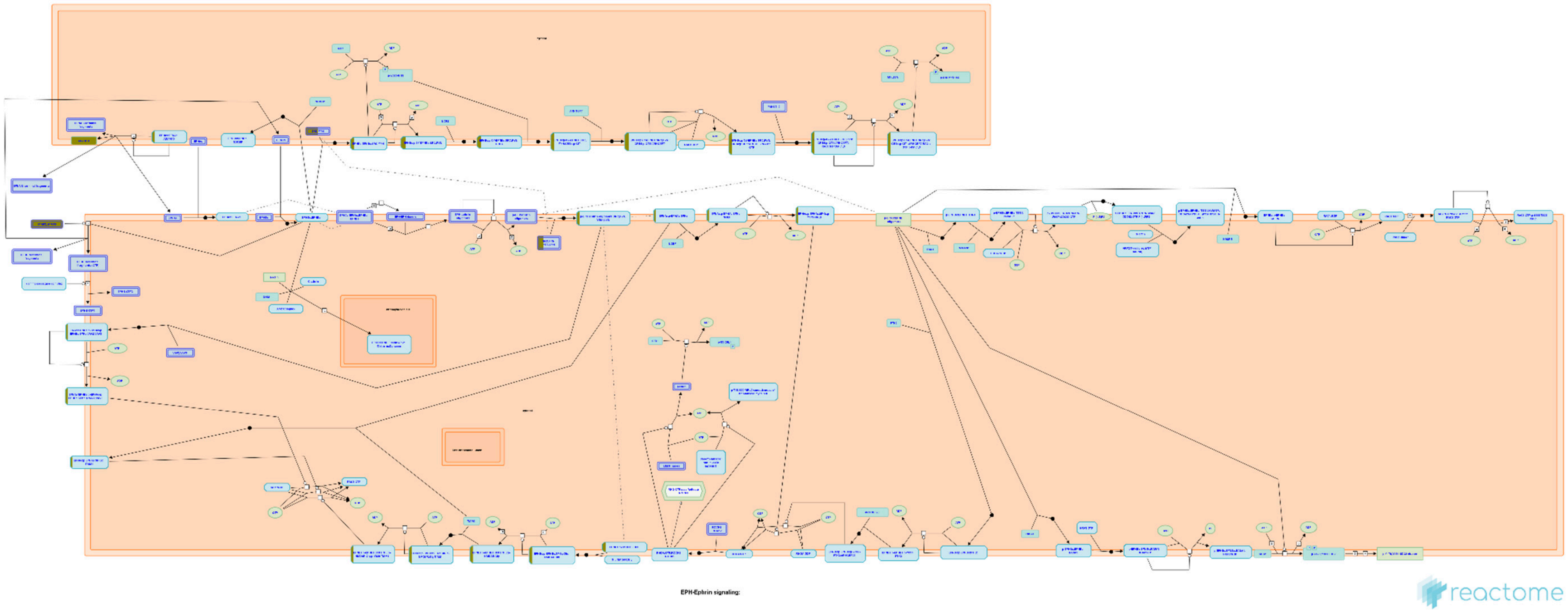

Figure S13. Affected proteins in the EPH-ephrin mediated cell repulsion pathway from the Reactome database (R-HSA-2682334).

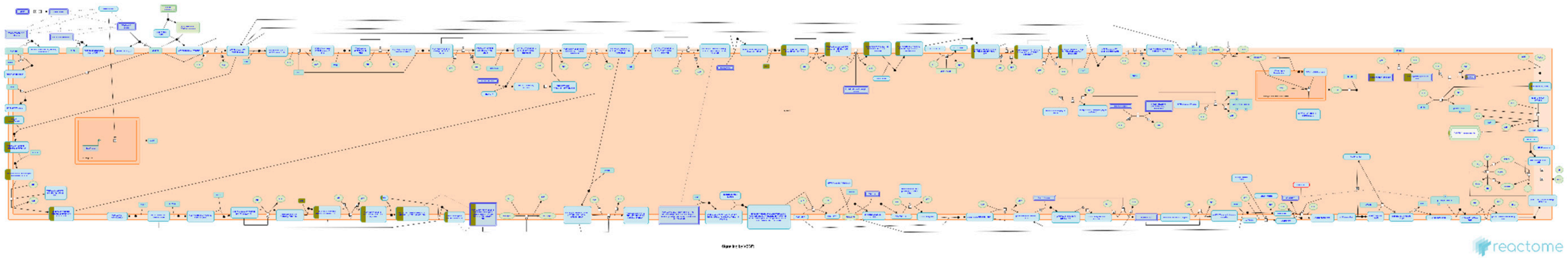

Figure S14. Affected proteins in the VEGFA-VEGFR2 pathway from the Reactome database (R-HSA-194138).

Table S4. Identified protein targets from protein-protein interaction network analysis.

Protein	PDB ID	RMSD (Å)	Binding Sphere Generation
FYN	2DQ7	1.017	Volume of complexed staurosporine
PDGFRB	3MJG	1.086	Grid-search and eraser algorithm (largest volume)
PIK3R1	4JPS	1.041	Volume of complexed 1LT inhibitor
ITGB3	6MK0	1.125	Volume of bound TDI-4161 antagonist
PDGFRA	6JOK	0.965	Volume of complexed sunitinib

Table S5. CDOCKER energy (in kcal/mol) of protein-ligand complexes.

Ligand	Form No.	FYN (PDB: 2DQ7)	PDGFRB (PDB: 3MJG)	PIK3R1 (PDB: 4JPS)	ITGB3 (PDB: 6MK0)	PDGFRA (PDB: 6JOK)
**1**	—	-2.15	-5.59	-1.10	6.11	1.26
**5**	1	-20.26	28.25	-23.29	-2.65	0.30
	2	-4.49	19.55	-7.93	15.29	21.59
	3	-17.41	24.42	-17.62	8.38	—
	4	-26.02	20.07	-27.64	-2.51	-21.61
	5	-11.90	30.26	-9.91	16.00	6.86
**15**	—	-8.28	-14.70	-18.24	-6.95	-23.09
**23**	—	-11.44	-11.08	-9.71	-5.37	-2.95
**24**	—	12.94	6.41	13.57	9.00	11.01
**28**	1	-47.51	-46.48	-50.71	-37.50	-40.24
	2	-34.69	-38.22	-45.94	-28.14	-34.44
**32**	1	-25.84	-22.65	-29.99	-13.26	-21.64
	2	-27.64	-26.98	-28.06	-15.65	-19.77
	3	-6.58	-10.16	-18.29	-0.44	-8.44
	4	-30.73	**-30.81**	-44.97	-20.86	**-36.41**
	5	-11.76	-17.67	-32.91	1.84	-11.48
	6	**-33.41**	-28.53	**-45.75**	**-21.64**	-33.88
	7	13.84	24.95	5.78	31.70	19.11
**36**	1	-19.65	-20.24	-16.16	-16.68	-14.29
	2	-8.38	-10.24	-7.03	-4.21	-8.91
**37**	—	-3.90	-8.17	-5.53	-4.07	-1.66
5FU [1]	1	-18.41	-24.21	-23.30	-18.89	-21.91
	2	-9.18	-13.65	-9.73	-10.26	-9.03
	3	-4.28	-10.22	-7.76	-11.31	-6.07
	4	-4.42	-10.69	-7.96	-10.65	-7.45
	5	-6.70	-14.10	-11.74	-11.37	-9.24
	6	-5.10	-10.45	-10.52	-11.14	-9.14
	7	-6.24	-10.42	-7.07	-8.28	-5.95
	8	-2.48	-9.34	-3.90	-9.28	-4.38
	9	-5.45	-10.36	-10.66	-6.48	-6.63
RGD [2]	—	-73.90	—	-73.88	-64.70	—
ALP [2]	1	-20.66	-27.90	-23.35	-20.76	-19.80
	2	-16.07	-18.86	-15.16	-17.47	-14.62
	3	2.49	-3.00	-1.92	-1.39	4.54
	4	-18.81	-28.69	-23.48	-16.13	-19.42
	5	-22.05	-24.07	-23.35	-14.86	-15.48
	6	3.36	-6.93	-0.29	5.26	4.09

Ligand	Form No.	FYN (PDB: 2DQ7)	PDGFRB (PDB: 3MJG)	PIK3R1 (PDB: 4JPS)	ITGB3 (PDB: 6MK0)	PDGFRA (PDB: 6JOK)
	7	2.81	-5.68	2.15	13.25	1.88
NIN [2]	1	-28.65	-1.13	-26.22	-35.48	-9.61
	2	0.50	9.99	2.75	-13.38	8.20
	3	-4.38	1.62	-6.84	-18.52	5.03
	4	-2.46	-5.15	0.71	-16.71	—
	5	-1.81	29.48	1.28	-12.89	12.10
	6	-20.65	-18.18	-23.24	-21.03	-18.00
	7	2.84	11.01	3.50	9.44	13.40
	8	-1.98	15.99	-6.79	-1.49	18.37
	9	4.14	3.87	3.29	4.94	12.49
	10	-0.12	10.57	3.61	3.10	5.19
PON [2]	—	-20.05	-8.81	-16.99	-15.11	-5.12
SUN [2]	1	92.90	—	113.16	98.34	119.43
	2	92.90	—	113.16	98.34	119.43
	3	92.14	—	107.85	94.88	118.46
	4	-5.39	-16.31	-9.29	-13.15	-2.74
	5	-12.97	-17.91	-11.80	-15.37	-10.66
	6	-6.86	-10.37	-5.32	-13.71	-6.08
	7	24.04	17.46	25.86	20.58	21.81
	8	22.11	16.23	19.12	16.46	15.97
	9	-8.55	-15.99	-12.92	-5.58	-8.28
	10	-10.66	-17.62	-16.10	-2.77	-10.41

Abbreviations: 5FU, 5-fluorouracil; RGD, [RGD-ChgE]-$CONH_2$; ALP, alpelisib; NIN, nintedanib; PON, ponatinib; SUN, sunitinib. [1] positive control of *in vitro* DU-145 cytotoxicity tests; [2] known inhibitors of protein targets.

References

1. Lee, Y.H.; Kim, B.; Kim, S.; Kim, M.S.; Kim, H.; Hwang, S.R.; Kim, K.; Lee, J.H. Characterization of Metabolite Profiles from the Leaves of Green Perilla (Perilla Frutescens) by Ultra High Performance Liquid Chromatography Coupled with Electrospray Ionization Quadrupole Time-of-Flight Mass Spectrometry and Screening for Their Antioxidant Properties. *J Food Drug Anal* **2017**, *25*, 776–788, doi:10.1016/J.JFDA.2016.09.003.

2. Fujita, T.; Terato, K.; Nakayama, M. Two Jasmonoid Glucosides and a Phenylvaleric Acid Glucoside from Perilla Frutescens. *Biosci Biotechnol Biochem* **1996**, *60*, 732–735, doi:10.1271/BBB.60.732.

3. Chen, J.; Guo, L.; Yang, G.; Yang, A.; Zheng, Y.; Wang, L. Metabolomic Profiling of Developing Perilla Leaves Reveals the Best Harvest Time. *Front Plant Sci* **2022**, *13*, doi:10.3389/FPLS.2022.989755.

4. Zhang, J.L.; Yan, R.J.; Yu, N.; Zhang, X.; Chen, D.J.; Wu, T.; Xin, J.G. A New Caffeic Acid Tetramer from the Dracocephalum Moldavica L. *https://doi.org/10.1080/14786419.2017.1359168* **2017**, *32*, 370–373, doi:10.1080/14786419.2017.1359168.

5. Jeong, J.H.; Park, H.J.; Chi, G.Y.; Choi, Y.H.; Park, S.H. An Ethanol Extract of Perilla Frutescens Leaves Suppresses Adrenergic Agonist-Induced Metastatic Ability of Cancer Cells by Inhibiting Src-Mediated EMT. *Molecules* **2023**, *28*, 3414, doi:10.3390/MOLECULES28083414/S1.
